# Impact of mouse contamination in genomic profiling of patient-derived models and best practice for robust analysis

**DOI:** 10.1186/s13059-019-1849-2

**Published:** 2019-11-11

**Authors:** Se-Young Jo, Eunyoung Kim, Sangwoo Kim

**Affiliations:** Department of Biomedical Systems Informatics and Brain Korea 21 PLUS Project for Medical Science, Yonsei University College of Medicine, Seoul, 03722 South Korea

**Keywords:** Benchmark, Patient-derived model, Genomic analysis, Mouse contamination, Best practice, Read filtering

## Abstract

**Background:**

Patient-derived xenograft and cell line models are popular models for clinical cancer research. However, the inevitable inclusion of a mouse genome in a patient-derived model is a remaining concern in the analysis. Although multiple tools and filtering strategies have been developed to account for this, research has yet to demonstrate the exact impact of the mouse genome and the optimal use of these tools and filtering strategies in an analysis pipeline.

**Results:**

We construct a benchmark dataset of 5 liver tissues from 3 mouse strains using human whole-exome sequencing kit. Next-generation sequencing reads from mouse tissues are mappable to 49% of the human genome and 409 cancer genes. In total, 1,207,556 mouse-specific alleles are aligned to the human genome reference, including 467,232 (38.7%) alleles with high sensitivity to contamination, which are pervasive causes of false cancer mutations in public databases and are signatures for predicting global contamination. Next, we assess the performance of 8 filtering methods in terms of mouse read filtration and reduction of mouse-specific alleles. All filtering tools generally perform well, although differences in algorithm strictness and efficiency of mouse allele removal are observed. Therefore, we develop a best practice pipeline that contains the estimation of contamination level, mouse read filtration, and variant filtration.

**Conclusions:**

The inclusion of mouse cells in patient-derived models hinders genomic analysis and should be addressed carefully. Our suggested guidelines improve the robustness and maximize the utility of genomic analysis of these models.

## Background

Patient-derived models (PDMs) serve as a way of preserving and amplifying cancer specimens of patients by providing in vivo or in vitro environments that allow the natural growth of cancer cells. The recent advent of various technologies for PDM construction, including patient-derived xenografts (PDXs), patient-derived tumor cell cultures (PDCs), and patient-derived organoids (PDOrg), has revolutionized translational cancer research by providing useful preclinical models of use in drug development [1, 2], drug screening [3–6], and a personalized co-clinical trials of cancer patients [7]. Particularly, next-generation sequencing (NGS) of these amplified primary tumors enabled robust measurement of genomic variants and gene expression changes under various conditions [8–11].

Genomic analysis of PDM samples, however, is more complicated than that of original primary tumor samples due to the rise of mouse-originating cells or tissues in the implanted specimen. Indeed, research has shown that resected PDX samples can harbor up to 70–80% murine DNA without a mistake in the separation process, mainly due to the infiltration of murine stromal cells [12–14]. Additionally, other in vitro models would also contain mouse genome fragments (e.g., fibroblasts in a co-cultured feeder layer or mouse sarcoma-derived Matrigel) [15, 16]. Subsequent next-generation sequencing of these samples would inevitably generate short reads of mouse DNA (mouse read, hereafter). Due to the genomic similarity between humans and mice [17], mouse reads are alignable to the human reference genome, which can cause multiple problems in standard genomic analysis: For example, once aligned, mouse-specific alleles in mouse reads are difficult to distinguish from true variants in human reads, resulting in false mutation calls. In transcriptome sequencing, mapping of cDNA mouse reads leads to aberrant gene expression profiles of cancer cells. Accordingly, researchers have lobbied continuing demands for efficient tools which deconvolute or remove murine effects in genomic analyses of PDM models [8, 14].

The removal of mouse reads has been primarily attempted computationally on NGS data. The simplest way to do this is to utilize the differential mappability of mouse reads onto the human and mouse reference genome. Thereby, reads that are mapped only to the mouse reference genome or are mapped better to the mouse than the human reference genome are filtered out. In the last few years, however, at least five computational tools [8, 18–21] have been developed to conduct the same task via different strategies and filtering criteria, and all have reported a satisfactory accuracy (~ 97.84%) in mouse read filtration. While these various solutions have increased the resources available to researchers, there are only a few benchmark studies on the effectiveness of these tools, and conclusions therefrom are inconsistent [8, 22, 23]. More fundamentally, it is still unclear as to whether the use of a filtration tool itself is essential or if steps other than the read filtration (e.g., variant blacklisting) are additionally required. To render an agreeable consensus, benchmark studies providing a comprehensive analysis of the true genome-wide effects of mouse reads, such as alignment landscape and gene- and locus-level vulnerability to contamination, on variant calling using a realistic dataset are needed.

Here, we report our benchmark results for the effectiveness of eight currently available mouse read filtering pipelines, reflecting their impact on genome analysis. To construct a realistic benchmark dataset, we directly sequenced mouse tissues that were processed by human exome target enrichment and further mixed with human reads at different rates, which allowed us to identify the quantity, mappability, and alignment landscape of mouse reads at a global level. To measure the impact of mouse reads on variant calling, we listed all mouse-specific alleles that could possibly cause mismatches (and ultimately false variants) in the aligned data and measured their sensitivity to contamination. Deeper analysis of the alleles led to the discovery of additional findings reflective of increased vulnerability in cancer genes and strain specificity, as well as the development of a robust measure for estimating contamination levels. Finally, pipelines were evaluated in terms of their efficiency in read filtering and reducing mouse-specific alleles, and the best practice pipeline was drawn, with additional suggestions for best output. We believe our study provides a basis for developing standards for genomic analysis of PDX and relevant patient-derived models.

## Results

### Construction of the benchmark dataset

**Fig. 1:**
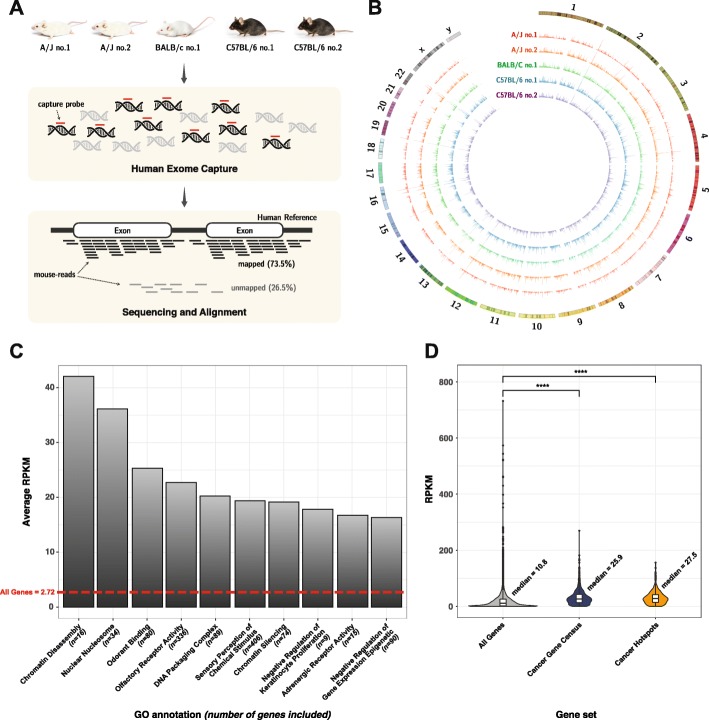
Impact assessment of mouse genome on human genome analysis. **a** Schematic overview of the data production to simulate mouse contaminated sample. **b** Coverage of five mouse samples on human genome reference (hg19). **c** Top ranked human functional gene sets enriched by mouse reads. Functional terms are annotated by Gene Ontology (GO). **d** Distributions of mouse read RPKM in all genes targeted by WES kit, Cancer Gene Census genes, and genes containing cancer hotspot mutations defined in cancer hotspots

Samples for the benchmark were obtained from fresh liver tissues from 5 mice (2 A/J, 1 BALB/c, and 2 C57BL/6 strains) (Fig. 1a). Tissues that passed initial quality control were prepared for NGS with human exome capture kits, with an average target depth of 200. Every raw NGS read (FASTQ) was marked with the mouse strain and replication numbers. To mimic mouse genome contamination in human samples and the exome-level sequencing thereof, public NGS data for 2 human lung cancers that were generated in the same manner as that for generating NGS data for the mouse samples (e.g., the same capture platform, version, and sequencing platform) were downloaded and mixed with raw mouse reads at 5 different rates (5%, 10%, 20%, 50%, and 80% of the total reads). The generation of each mixture was triplicated with different randomization seeds to remove downsampling effects. Finally, a total of 150 human-mouse mixture datasets (2 human × 5 mice × 5 mixture rates × 3 downsampling randomizations) were prepared for the analysis (see the “Methods” section and Additional file 1: Table S1).

### Impact of mouse contamination on genomic analysis

We first mapped the NGS reads from 5 pure mouse samples to the human genome reference to trace the mouse reads during alignment. Of the 117,239,374–126,090,704 mouse reads that were physically captured by human exome enrichment kit, 84,819,721–97,585,087 (75.1% on average) were mapped to the human reference genome (hg19) with a conventional read alignment process (BWA-MEM, default setting, see the “Methods” section). At a global level, these aligned mouse reads were evenly distributed across all human chromosomes, except the Y chromosome, with only slight differences among strains (Fig. 1b). The aligned mouse reads covered 49.0% of all human protein-coding regions, stretching across 10,277 RefSeq genes (out of 21,429; 48.0%). Moreover, these genes included 409 of 720 CGC (COSMIC Cancer Gene Census, Sanger Institute [24]) cancer genes for a coverage of 56.8%.

We further assessed gene-specific sensitivity to mouse reads. Based on a normalized read count (reads per kilobase per million (RPKM) mapped reads), genes of higher mappability to mouse reads could be rendered (Additional file 1: Figures S1 and S2, Additional file 2). Among them, 2822 (13.2%) genes were highly sensitive to mouse reads, with an average RPKM > 40; this corresponds to 20,000~30,000 mapped reads per average-sized gene (10~15 kb) in a typical 100× exome paired-end sequencing with a 100-bp read length. We also found that the top sensitive genes were associated with essential cellular functions such as chromatin structure, nucleosome, sensory receptors (Fig. 1c, Additional file 3), and many cancer genes including *CDH11* (cadherin11) and *SOX2* (sex-determining region Y) (Additional file 1: Figure S2B). For further analysis, we presumed that human cancer genes that tend to play a critical role in cellular proliferation and regulation would be more sensitive to mouse reads due to their lower tolerance to sequence variations and higher inter-species conservation. The RPKM distribution within all human and CGC genes, as well as cancer hotspot variant sites (cancer hotspots, Memorial Sloan Kettering Cancer Center [25]), reflected an increased mappability of mouse reads to cancer genes and hotspots (median RPKM 25.9 and 27.5 vs. 10.8), confirming our hypothesis (Wilcoxon rank-sum test *p* values of 2.46 × 10^−69^ and 1.90 × 10^−30^) (Fig. 1d). These results demonstrated that mouse reads, once included in the samples, are difficult to filter with standard alignment procedures and affect downstream genomic analysis, particularly for cancer genes.

### Characteristics of human genome-aligned mouse alleles

**Fig. 2:**
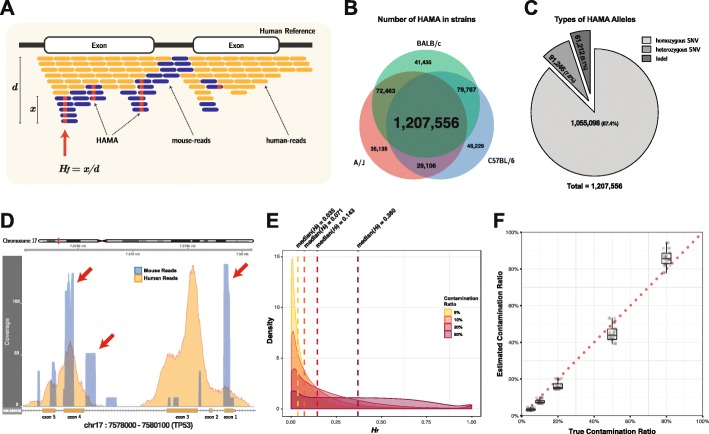
Schematic overview and characteristics of human genome-aligned mouse allele (HAMA)**. a** Definition of HAMA and their allele frequency. *H*_*f*_ is defined as *x*/*d*, where *d* is the total depth of given position, and *x* is the depth of all allele from mouse reads. **b** Common and Strain-specific HAMA. **c** Types of HAMA alleles. HAMA alleles consist of 87.37% homozygous SNVs, 7.56% heterozygous SNVs, and 5.07% indels. If any of the five mouse samples were reported as heterozygous SNVs, we counted as heterozygous SNVs. **d** Example of genomic regions that contains high-risk HAMAs (50% contamination ratio, TP53, exons 1–5). The coverage of human reads colored in yellow and mouse reads in blue. Red arrows indicate the genomic regions where the coverage of mouse reads dominates that of human reads. **e** Distributions of *H*_*f*_ for all HAMA sites in four different global contamination levels (5%, 10%, 20%, and 50%). Median *H*_*f*_ is denoted by dotted lines. **f** Estimation results of all in silico contaminated dataset based on the linear regression of median *H*_*f*_. Red dotted line indicates the perfect estimation line

A major problem with variant analysis of PDM stems from the fact that mouse-specific alleles look like somatic mutations in the samples. While the locations of these alleles and their corresponding human loci are difficult to identify at the reference genome level due to a complex homolog structure, more practical assessment can be achieved in the read alignment step. Among mouse reads, we defined mouse alleles that were alignable to the human genome as human genome-aligned mouse alleles (HAMAs) (Fig. 2a). Although the actual list of HAMAs differed according to the mouse strain, sequencing protocol (e.g., read length, capture efficiency), and alignment tool, we assumed that impactful HAMAs would be repeatedly observed when applying conventional protocols.

In our benchmark setting, a total of 1,602,035 HAMAs were observed from the 5 mouse samples, 1,207,556 of which were shared by all mice (common HAMA). This corresponded to the 3.28% of all bases covered by the mouse reads. Meanwhile, 35,137, 41,435, and 46,229 strain-specific HAMAs were identified in A/J, BALB/c, and C57BL/6 mice, respectively, showing decreased mismatches between A/J and humans (Fig. 2b and Additional file 1: Table S2). The entire list of common HAMA is available in Additional file 4.

Individual HAMAs pose distinctive risks of contamination reflected in the variant allele frequency (VAF) of the allele together with the number of human reads aligned at the site. Thus, we defined *H*_*f*_ (HAMA allele frequency) as the variant allele frequency of a HAMA (Fig. 2a). For each HAMA site, *H*_*f*_ value is determined by 3 major factors: (i) mappability of HAMA-containing mouse reads, (ii) mappability of human reads at the site, and (iii) the overall contamination level. Thus, HAMAs with good mouse read, but low human read mappability, would have larger *H*_*f*_ values and would pose a greater chance of being called as (false) mutations. In the actual calculation of *H*_*f*_, we used the read counts of mouse reads from the benchmark dataset for (i) and the mean read depth of 125,748 human whole-exome sequencing from the gnomAD database [26] for (ii). By changing the mixture ratio of (i) and (ii), we could calculate *H*_*f*_ values at different contamination levels (iii) (see the “Methods” section for details).

The overall distributions of common 1,207,556 *H*_*f*_ values at 4 different contamination levels (5%, 10%, 20%, and 50%) varied greatly (Fig. 2e). For a given contamination level *α*, the *H*_*f*_ of *α* suggests that the mappability of a mouse read is similar to that of a human read at the HAMA. For most cases, *H*_*f*_ would be lower than *α* due to the reduced mappability of mouse reads, which was observed in a positive-skew distribution and in observed median *H*_*f*_ values of 3.7%, 7.4%, 14.8%, and 38.9% for *α* values of 5%, 10%, 20%, and 50%, respectively. However, we found a substantial number of HAMAs (454,438 out of 1,207,556; 37.6%) with > *α* were also present, suggesting that these HAMAs are highly sensitive to contamination. Further investigation confirmed that these regions are poorly targeted in whole-exome sequencing (WES), but more preferentially aligned by mouse reads (Fig. 2d, red arrows). To represent the sensitivity of HAMAs to contamination, we finally defined *H*_*c*_ (HAMA allele frequency coefficient) as the expected *H*_*f*_ per 1% overall contamination. Using *H*_*c*_, we can explicitly quantitate the intrinsic risk of HAMAs and predict the expected *H*_*f*_ as follows:

1 $$ {H}_f=\alpha {H}_c, $$

where *α* is the global contamination level of a sample. We defined 454,438 HAMAs with *H*_*c*_ ≥ 1 as high-risk HAMAs. Similarly, low-risk HAMAs are defined as *H*_*c*_ < 1 (see Additional file 4 for the full list of HAMA and their *H*_*c*_ values).

Deducing from Eq. (1), a global contamination level can be also estimated by *H*_*f*_ and *H*_*c*_ as follows:

2 $$ \alpha ={H}_f/{H}_c $$

As *H*_*c*_ is HAMA-intrinsic, measuring only *H*_*f*_ gives an estimate of *α*. From the benchmark dataset, we found that the median of *H*_*f*_ is linearly correlated with *α* with an average *H*_*c*_ of 0.7519 (Additional file 1: Figure S3). Hence, the contamination level can be calculated in a single sample as follows:

3 $$ \alpha =\mathrm{median}\left({H}_f\right)/0.7519 $$

Applying (3) to the 150 single samples in the benchmark dataset (5–80% contamination) showed a good estimation of the global contamination levels within a small error size (0.4–2%, 95% CI) (Fig. 2f). Although a slight under- and overestimation in low-to-medium (< 50%) and high (80%) contamination levels imply more complex (e.g., non-linear) characteristics, we expect *H*_*f*_ to be a simple, convenient, and instant estimator of global contamination of PDM samples.

### Impact of mouse alleles in variant calling

**Fig. 3:**
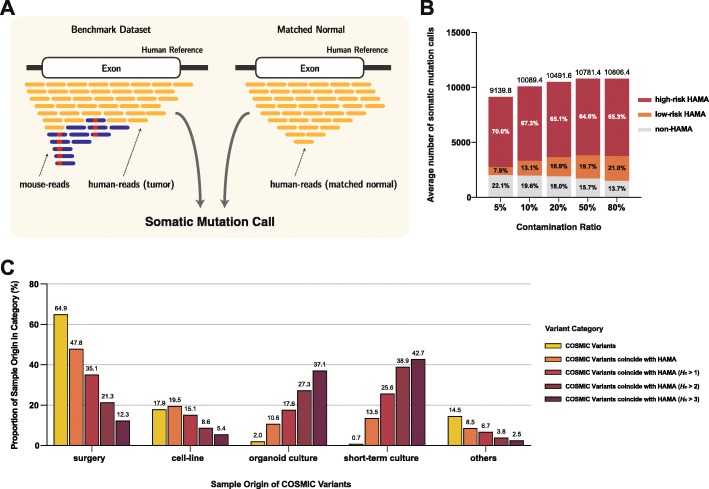
Impact of mouse alleles on SNV calling. **a** A schematic overview of somatic mutation calling on benchmark dataset. **b** Number of HAMAs and their ratios in somatic mutation call. Numbers are averaged from all the benchmark set. **c** Number of studies that have reported COSMIC confirmed variants with specified sample origins. Sample origin notation follows the classification of COSMIC database

Next, we sought to determine whether HAMAs are detectable as somatic mutations (Fig. 3a). For the analysis, we applied a conventional pipeline for somatic mutation detection (the GATK best practice [27], see the “Methods” section) to human cancer sequencing data in which 4 different amounts of mouse reads were mixed at global contamination levels of 5%, 10%, 20%, 50%, and 80%. The numbers of mutation calls were far larger than the general tumor samples, with a positive correlation with the contamination levels (9140, 10,089, 10,492, 10,781, and 10,806 in 5%, 10%, 20%, 50%, and 80%, respectively). Of them, ~ 70% of the calls overlapped with high-risk HAMA sites for all contamination levels, implying that high-risk HAMAs are major sources of false somatic mutation calls (Fig. 3b, red color). On the other hand, the portions of low-risk HAMA calls were substantially smaller and varied depending on contamination levels (7.9%, 13.1%, 16.9%, 19.7%, and 21.0% of all calls in 5%, 10%, 20%, 50%, and 80% contamination level, respectively Fig. 3b, orange color). As a minimum variant allele frequency is required to be called as somatic mutations (in general, 5–10% [28]), low-risk HAMAs become callable only above a certain level of contamination. Therefore, we conclude that high-risk HAMAs are of primary concern in terms of variant calling.

Moving forward, we assessed if false somatic mutations derived from HAMAs are included in a public database. Of 1,207,556 common HAMAs, 103,481 (5.6%) were present in the most recent version of COSMIC (version 88, March 2019), accounting for 2.14% of all 4,843,731 confirmed variations. As the COSMIC database collects and confirms somatic mutations from independent studies, we further assessed their evidential basis. Out of 6,842,627 studies that reported COSMIC somatic mutations, 2,453,873 (35.9%) specified sample origins without ambiguity (e.g., “NS”, see the “Methods” section). Of them, 46,540 reported HAMA variants. We found a clear difference in the proportion of sample origins between HAMA and other COSMIC variants (Fig. 3c and Additional file 1: Figure S4). Regarding all COSMIC variants, most of the supporting studies specified their sample origins as surgery (64.9%) (Fig. 3c, yellow bars). This proportion was decreased in HAMA variants (47.8%) and more decreased as considering only high-risk HAMAs (35.1, 21.3, and 12.3% in HAMA with *H*_*c*_ > 1, 2, and 3, respectively). A similar change in the proportion was observed in cell line studies. On the other hand, the proportions of studies from organoid and short-term culture were remarkably higher in high-risk HAMAs (up to 37.1 and 42.7%, respectively) compared to those in all COSMIC variants (2.0 and 0.7%, respectively). These results indicated that HAMAs, particularly high-risk HAMAs, are likely to be reported as cancer somatic mutations in studies of cultured samples.

### Effects and comparison of current methods for mouse read filtration

As shown in the series of analyses in this manuscript, filtering mouse reads is crucial for accurate genomic analysis of PDM data. For this reason, several study groups have designed tools which deconvolute mouse reads in NGS data obtained from PDMs. Currently, there are five available tools: BBsplit [18], Xenome [19], Bamcmp [8], Disambiguate [20], and XenofilteR [21] (Additional file 1: Figure S5).

BBsplit and Xenome take FASTQ files and compare sequence similarities of raw reads to both the human and mouse reference genomes in order to extract human origin reads. Bamcmp, Disambiguate, and XenofilteR take two BAM files that are mapped to the human and mouse reference genomes and use mapping quality to discard reads that are mapped better to the mouse genome. While the general approaches of these tools are overall quite similar, user-generated changes in the parameters, including cutoff values and strictness, may result in different accuracies. In addition to the five tools above, three simple methods can also be applied to filer mouse reads. One involves the use of a human-mouse concatenated reference (ConcatRef, hereafter) to exploit the judgment of an alignment algorithm (e.g., BWA-MEM) in order to find the best place for mapping NGS reads. In doing so, reads that are better mapped to the human reference side (over the mouse side) are thought to be human reads. Two others involve aligning reads to human and mouse reference genomes independently (DualRef), and reads that are mapped to the mouse are filtered out: One discards all mouse genome-aligned reads (DualRef-S; DualRef with strict filtering); this was named “strict filtering” in [21]. The other discards only mouse genome-aligned reads with no mismatch (DualRef-L; DualRef with lenient filtering) (see the “Methods” section for details).

We applied all eight methods (the five tools and three simple methods) to our benchmark dataset to evaluate their performance in two different categories: (1) accuracy of read filtering and (2) reducing variant allele frequencies of HAMAs (*H*_*f*_) (Additional file 1: Table S1, see the “Methods” section for detailed benchmark procedures). For (1), the remaining and filtered reads were traced after the application of the eight methods. We defined sensitivity as the proportion of mouse reads that were correctly filtered out and specificity as the proportion of human reads that remained after filtration. *F*-score was calculated as a balanced measure of sensitivity and specificity. For (2), *H*_*f*_ values were measured after filtrations and were compared with unfiltered values.

**Fig. 4:**
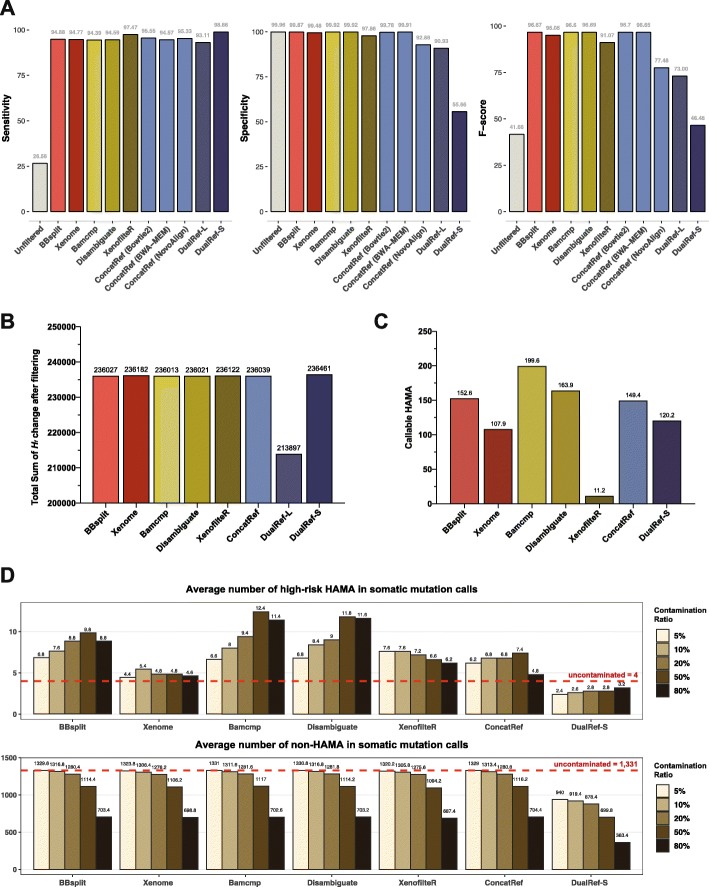
Performance of eight filtering methods measured in the benchmark dataset. **a** Sensitivity, specificity, and *F*-scores of eight filtering methods in terms of mouse read filtration. **b** Total sums of *H*_*f*_ reduction after filtration. **c** Numbers of callable HAMA (*H*_*f*_ > 5%, alternative allele count > 5) after filtration. **d** Numbers of mutation calls in high-risk HAMA and non-HAMA sites after filtration

Read filtering analysis confirmed a generally good performance of all methods except two dual reference methods (DualRef-S and DualRef-L) (Fig. 4a). In terms of sensitivity, all methods marked > 93%, wherein DualRef-S and XenofilteR showed the best mouse read filtration rate. However, DualRef-S marked very low specificity (55.7%) by losing almost half of human reads. Except for the DualRef-L (90.9%) and XenofilteR (97.9%), all tools marked specificity of ≥ 99.5%. While there is a clear trade-off between sensitivity and specificity, four methods Disambiguate, BBsplit, ConcatRef, and Bamcmp showed the best balanced measure. However, XenofilteR would be useful where strict mouse read filtering is required.

With the unexpected performance of the simple ConcatRef method, which was comparable to that of the five tools, we further tested its overall accuracy when applying different alignment algorithms. Among Bowtie2 [29], BWA-MEM [30], and NovoAlign [31], Bowtie2 showed the best performance with an *F*-score of 96.7, which was highest among all eight methods (Fig. 4a, blue bars). Therefore, disregarding other features of speed and ease of use, which can be important to users, we concluded that a simple implementation of ConcatRef works as effectively as the top specialized tools.

In the allele frequency-based evaluation, all tools successfully reduced *H*_*f*_ (Fig. 4b and Additional file 1: Figure S6). The sums of total *H*_*f*_ reductions were similar (236,031–236,461) except DualRef-L (213,897). These numbers correspond to 17.7–19.58% reduction of allele frequency for each HAMA site. We further examined the number of HAMA sites that might be callable by mutation calling pipelines. Assuming *H*_*f*_ of 5% and alternative allele count of 5 as the minimum conditions for mutation call [32], XenofilteR left the fewest number of callable HAMAs (7.8 on average), followed by Xenome (77.6), DualRef-S (87.7), and ConcatRef (113.1) (Fig. 4c). In contrast to read filtering measure, minimization of *H*_*f*_ values are achieved by high sensitivity (filtering mouse reads) than high specificity (conserving human reads), except DualRef-S (too low specificity, 55.66%). Finally, we applied a somatic mutation calling pipeline to the filtered BAM files from eight methods (Fig. 4d). Except for DualRef-S, all 7 methods dramatically reduced the number of calls in high-risk HAMA sites (3 to 12 calls), compared to unfiltered data (7121 to 9088 calls, Fig. 3b) and to uncontaminated data (4 calls, Fig. 4d, top, red lines). Among them, DualRef-S, Xenome, XenofilteR, and ConcatRef showed robust performance even in high contamination ratio (50%), while DualRef-S also removed a large number of non-HAMA variants (Fig. 4d, bottom). Therefore, we conclude the Xenome, XenofilteR, and ConcatRef are the top 3 filtering methods in terms of variant calling.

### Additional strategies for better analysis

As filtration of mouse reads is only one part of the analysis pipeline, we sought to determine if additional optimization can be made in other parts thereof, including read alignment, variant filtration, and other pre- and post-processing steps. Here, we posed and tested three additional strategies that may be applicable to improve the quality of the pipeline.

The first potential approach is to build a blacklist of genomic loci that are frequently called as variants. Even after mouse read filtration, we discovered that 7–151 HAMA sites remained callable (Fig. 4c). To test if blacklisting of HAMA sites efficiently removes the remaining false variants, we applied 2 variant filtration approaches: (1) filtration of all common HAMAs (strict blacklisting) and (2) filtration of only high-risk HAMAs (*H*_*c*_ > 1) (lenient blacklisting). We observed a mean of 2.9 mouse-derived false variants in somatic mutation calls using Mutect2 even after applying the filtering methods. Both strict and lenient blacklistings were almost equally satisfactory in their ability to remove the remaining false variants, leaving approximately 0.7 and 0.8 false variants, respectively. However, strict blacklisting lost more than twice of the human-derived true variants than lenient blacklisting (11.5 vs. 4.8 variants, respectively) (Additional file 1: Figure S7, Additional file 1: Table S3). The choice of blacklist types can be dependent on the purposes; however, we conclude that the lenient blacklisting can be applied generally with a minimum risk.

Another strategy involved inference and estimation of global contamination levels, the feasibility of which we showed using *H*_*f*_. Estimated contamination levels are more useful when DNA and transcriptome sequencing data are generated from the same PDM sample, as gene expression profiles are easily disrupted by the inclusion of mouse cells in a sample. We expected that the inferred contamination level could be further used in gene expression analysis tools for mixed samples [33, 34]. We also expect that we could apply the inferred contamination level in adjusting strictness for variant filtering, as more low-risk HAMAs can be present in highly contaminated samples. While the exact cutoff value for variant filtering strategy needs more investigation, our benchmark results show that even in 50% contamination, lenient blacklisting outperformed strict blacklisting (Additional file 1: Table S3). Again, however, strict blacklisting can be an option in high-contamination samples (e.g., > 50%) depending on the study design.

The final strategy relied on the use of a strain-specific reference genome in the alignment. Since the current mouse reference genome (GRCm38 or mm10) has been built based on the C57BL/6 strain [35], we assumed that alignment on reference genomes of matching strains [36] would increase the mappability of mouse reads and further improve the filtration efficiency. In the test with the A/J and BALB/c reference genomes and the benchmark datasets thereof, however, we could not find sufficient evidence for the hypothesis, with the same specificity and even ~ 1% reduction in sensitivity (Additional file 1: Figure S8). Further investigation identified that the current strain-specific genomes are basically the same with the reference genome with only a substitution of one or two chromosomes with shorter versions (chr13 in A/J and chr7 and 12 in BALB/c [37]). Therefore, we conclude that the use of a strain-specific reference genome is not beneficial, at least currently.

### Best practice for analysis of PDM sequencing

**Fig. 5:**
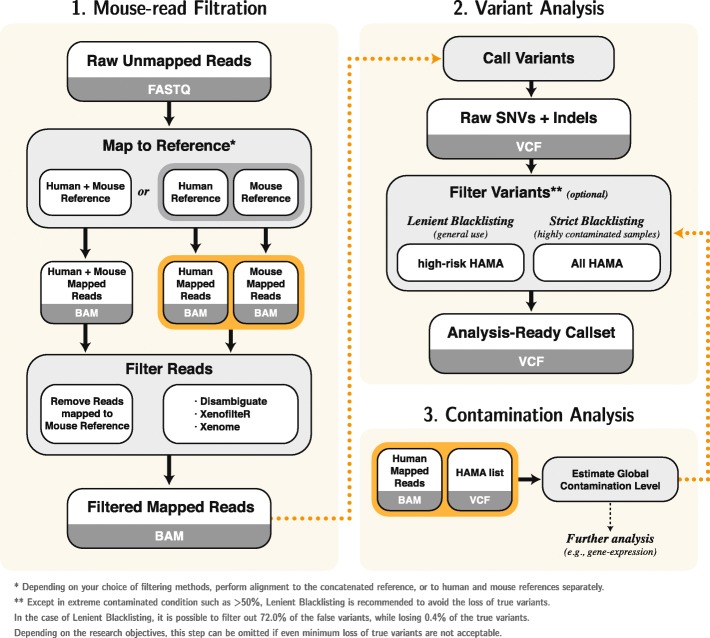
Best practice for analysis of PDM sequencing. A robust workflow to analyze human genome data contaminated by mouse genome. ConcatRef, Disambiguate, and XenofilteR are the best suggested filtering method for general purpose. Alternatively, Xenome, XenofilteR, and ConcatRef are also recommended for SNV analysis. After applying a filtering method, further filtering can be optionally achieved by blacklisting using HAMA list. Estimation of contamination ratio can be used as an indicator of whether strict or lenient blacklisting should be applied

Based on the benchmark results, we suggest that the best practice for genomic analysis of PDM sequencing (Fig. 5) ought to consist of (1) alignment to human and mouse reference genomes, (2) estimation of the contamination level, (3) application of mouse read filtering methods, and (4) variant filtration using blacklists. Reference genomes can be prepared either as two separate genomes (human and mouse) or in a concatenated form (human plus mouse), depending on the filtration method used. Before filtration, the global contamination level can be inferred from a median of *H*_*f*_ values for common HAMA sites aligned to the human genome and used for other independent analyses (e.g., gene expression). For mouse read filtration, all methods except DualRef-S and DualRef-L are generally useful. However, if read filtration itself is the final goal, ConcatRef, Disambiguate, and BBsplit are the top-performing methods, while Xenome, XenofilteR, and ConcatRef are the better options for preventing false somatic mutations. After variant calling, HAMA blacklisting can be optionally applied to the call set. In general, high-risk HAMA sites can be filtered from the called somatic mutations (lenient blacklisting), where filtration of all common HAMA sites can be optionally applied (strict blacklisting) in highly contaminated samples. Although a cutoff value of 50% is proposed for the choice of blacklisting method, we would like to note that this is still arbitrary as we did not observe the point where strict blacklisting starts to be more beneficial. Note that blacklisting may discard ~ 1% of true variants and can be omitted in some studies that require high sensitivity such as the discovery of new functional mutations. We suggest that any called variants that overlap HAMA should be reviewed before proceeding to further analysis step.

## Discussion

Constructing a gold standard is the first key step for high-quality benchmark studies. In this study, we tried to realistically simulate contamination by processing mouse genomes with a human capture platform, followed by alignment to the human genome. In the in silico mixture, we used human lung cancer sequencing data to analyze on a frequently targeted disease model in PDM [38]. Nevertheless, we assume that the choice of human sequencing data would not affect the overall result, due to the lack of tissue and disease specificity in genomic DNA sequences. On the other hand, the use of multiple strains and replications in data generation is a strong point of our study, although consideration of the number of samples is warranted. It is, however, difficult to define an optimal number of samples for obtaining a gold standard for genomic analyses, as genome sequences are believed to be nearly identical among tissues and quality-controlled, commercial mice. That said, increases in data size are usually beneficial. Nonetheless, even in the same sample [39], there might be risks for accidental deviations (e.g., low sample quality, low sequencing coverage, and allele dropout) in part of a benchmark set. Although we tried to avoid these risks by aggregating sample data and only using commonly shared alleles (e.g., common HAMAs), caution must be taken when using strain- or individual-specific alleles, especially for BALB/c mice, for which we only included sequencing data from one mouse. We expect that subsequent studies attempting to reproduce our results will solidify the consensus.

In the suggestion of the best practice, we did not specify a single tool for mouse read filtration due to the similarities in their accuracies, as features other than performance are also important in practice. Comparisons of tools in terms of language, features, and running speed are available in Additional file 1: Table S4. In this regard, the user might find Disambiguate favorable due to its good speed and convenient running procedures. ConcatRef is also a good method, once a concatenated reference is prepared, as the entire process ends with an alignment step. XenofilteR can be a good choice for users who are familiar with the R language and also showed good speed. We had a few problems in testing Xenome due to memory-related errors and a relatively low speed, which might not occur with other users. Overall, we recommend users to test the individual tools that are included in the best practice to find one that best fits their environment.

As RNA-seq is another prominent part in PDM sequencing, similar analyses are urgently required to render the best practice. We would like to note that benchmarking for transcriptome analysis is far more complicated due to the disease, tissue, and cell specificity of gene expression, as well as their stochastic nature. Therefore, the construction of benchmark datasets that consist of multiple tissues and a number of replicates will be important. Confining datasets to a specific tissue (e.g., fibroblast) can alleviate complexity and will be a starting point for rendering best practice. We also expect that the use of HAMA will be a good resource in the development of new tools for analyzing both DNA and RNA sequencing data, by cataloging homo- and heterozygous mouse alleles.

Finally, as sequencing and relevant laboratory technologies are rapidly evolving, genomic analysis of PDMs could be further improved from the suggested best practice. The most fundamental preprocessing can be done before sequencing, by directly separating mouse cells from samples. Fluorescence-activated cell sorting (FACS) or immunomagnetic separation (IMS) on mouse-specific antibodies can be feasible methods, while problems in time, cost, and lack of applicability (e.g., formalin-fixed paraffin-embedded samples) must be resolved beforehand. Sequencing with a longer read length can be utilized in assessing relationships between sample origins of two or more variants, such as by haplotype phasing [40, 41] or chromatin-level aggregation (e.g., somatic co-mutation hotspots [42]). Accumulation of information about human- and mouse-specific variants will also lead to novel algorithms, such as machine-learning-based deconvolution. In any form, cutting-edge technologies must be considered continuously for integration to the best practice to guarantee the most reliable analysis of PDM samples.

## Conclusions

By constructing a mouse tissue-driven benchmarking dataset, we confirmed that the inclusion of mouse alleles strongly affects downstream genomic analyses and must be handled with specialized pipelines. We found that mouse-specific alleles can be aligned to widespread regions of the human genome and are causative of false somatic mutations in PDM data. Comparison of eight available methods for mouse read filtering showed relatively small gaps in the performances thereof but identified a set of best tools. In addition to read filtering, we rendered a best practice pipeline that consists of contamination level estimation and variant-level filtering using blacklists for improved efficiency in calling true variants.

## Methods

### Data acquisition and processing mouse reads

Actual sequencing of mouse DNA with human DNA capturing kit—SureSelect Human All Exon V5—has been performed to obtain raw reads of mouse DNA. Two A/J mouse samples, two BALB/c mouse samples, and two C57BL/6 mouse samples have been sequenced, and all samples except one BALB/c sample passed QC. Therefore, five sequenced data were used in this study. Using the sequencing data of mouse DNA captured by human DNA capturing kit, we performed alignment to the human reference (hg19) with BWA-MEM. All the arguments of BWA-MEM are set to default (mismatch penalty = 4, gap open penalty = 6, gap extend penalty = 1), which is recommended in well-known pipelines including GATK best practices and NIH’s GDC Documentation. If the mismatch penalty increases, roughly a large number of mouse reads can be filtered out with a single alignment step. However, adjusting the parameter is not recommended since it can cause the loss of human reads.

### Quantitative analysis of mouse reads in human genome reference

A BED file defining the captured region of SureSelect Human All Exon V5 has been obtained from the Agilent website and counted all the read per captured region from BAM files using GATK4 CollectReadCounts (ver. 4.1.1.0). These tables are annotated with the NCBI RefSeq Gene database, and the read counts were grouped by gene using an in-house python script to count the number of reads per gene.

### Preparation of in silico mouse contaminated data

We generated hypothetical in silico mouse contaminated sample with TCGA human lung cancer WES data (TCGA-67-3771-01A-01D, TCGA-73-4658-01A-01D) and actual mouse WES data as described above (A/J no.1, A/J no.2, BALB/c no.1, C57BL/6 no.1, C57BL/6 no.2). Each mouse FASTQs are randomly downsampled to 5%, 10%, 20%, 50%, and 80%, regarding the human sample’s read count using seqtk [43] tool. Every downsampling is repeated three times using three random seeds. The human FASTQs were also downsampled to 95%, 90%, 80%, 50%, and 20% in the same manner of mouse samples and then combined with each complementary mouse sample (Additional file 1: Table S1).

### Identification of mouse-derived alleles aligned on human genome reference

The mouse reads aligned on human genome reference (hg19) prepared as above, are inputted to the GATK4 HaplotypeCaller (ver. 4.1.1.0) to call out all the SNVs and indels on the basis of a human reference (hg19). Next, common variants of all five mice samples are collected using an in-house Python script to exclude strain- or individual-specific variants. The entire list of common HAMA is available in Additional file 4.

### Calculation of general *H*_*f*_ values

A mean coverage file in gnomAD (ver. 2.1.1) was downloaded from the gnomAD website, from which we collected the mean coverage values for every HAMA position. Next, the mean coverage of five mice BAM files was calculated for every HAMA position. Finally, general *H*_*f*_ values at HAMA positions (*i)* were obtained using the following formula:

$$ {H}_f(i)=\frac{DP{(i)}_{\mathrm{mouse}}}{\left( DP{(i)}_{\mathrm{human}}+ DP{(i)}_{\mathrm{mouse}}\right)} $$

*DP*(*i*)_mouse_ represents the mean depth of 30 downsampled mouse samples on HAMA position *i*, and *DP*(*i*)_human_ represents the mean depth of 125,748 human samples registered in the gnomAD database.

Next, *H*_*c*_ (HAMA coefficient) of the given position (*i*) was calculated by dividing *H*_*f*_ by the contamination ratio *α*:

$$ {H}_c(i)=\frac{H_f(i)}{\alpha } $$

This coefficient (*H*_*c*_) represents the *H*_*f*_ value at a contamination level of 1%.

### Identification of HAMAs coincides with COSMIC variants

Using the HAMA list generated above, all the COSMIC (v88) variants that coincide with the HAMA list are collected from *CosmicCodingMuts.vcf* file. Next, the sample origins of the COSMIC IDs are collected from the *CosmicMutantExport.tsv* file. In this process, we excluded “NS,” “cultured-NOS,” “fixed-NOS,” “fresh/frozen-NOS.” and blank data to avoid ambiguity. The count results of all sample origins, which does not exclude anything, can be found in Additional file 1: Figure S4.

### Somatic mutation calling

Normal control samples in TCGA-67-3771-10A-01D were used as matched normals for tumor samples in TCGA-67-3771-01A-01D. Together with the benchmark dataset generated with TCGA-67-3771-01A-01D, these matched normal samples were input into GATK4 Mutect2 (ver. 4.1.1.0).

All parameters were set to default, and the gnomAD database (ver. 2.1.1) was applied to follow the GATK’s best practice for somatic calling.

### Application of filtration tools

#### Pre-alignment filtering tools—BBsplit and Xenome

The in silico contaminated dataset generated as above was input directly to each tool as FASTQ format. The resulting FASTQ files are aligned to GRCh37 human reference using BWA-MEM to make the final BAM file (Additional file 1: Figure S5A).

#### Post-alignment filtering tools—Bamcmp, disambiguate, and XenofilteR

The in silico contaminated dataset was aligned to human reference (hg19) and mouse reference (mm10) separately. These resulting BAM files are input to each tool as a pair to make the final BAM file (Additional file 1: Figure S5B).

#### Concatenated reference (ConcatRef)

The “concatenated reference” is prepared by merging human reference (hg19) and mouse reference (mm10) in series. The in silico contaminated dataset was aligned to this concatenated reference using BWA-MEM, and the final BAM file was completed by removing the reads that are aligned to mouse reference (mm10) (Additional file 1: Figure S5C). This process was reproduced with Bowtie2 and Novoalign for performance comparison.

#### Dual reference—lenient (DualRef-L)

First, the in silico contaminated dataset was aligned to mouse reference (mm10) and then collected the ID of the reads whose NM tag is 0. This process was performed using samtools, and the command line is as follows. Next, the final BAM is completed by removing the read with the corresponding read ID from the in silico contaminated BAM file aligned to the human reference (hg19) using picard FilterSamReads (Additional file 1: Figure S5D).

#### Dual reference—strict (DualRef-S)

In the same manner of DualRef-L, align the in silico contaminated dataset on mouse reference and collect the ID of all the reads that are successfully aligned on mouse reference. Next, remove all reads with the corresponding ID in the BAM file that is aligned to the human reference (Additional file 1: Figure S5E).

### Benchmark of known filtering tools

The in silico mixed sample dataset prepared in the mixture of two human, five mice, four mixture ratios, and three random seeds (Additional file 1: Table S1) was input to pre-alignment filtering tools (BBsplit, Xenome), post-alignment filtering tools (Bamcmp, XenofilteR, Disambiguate), simple implementation scripts (ConcatRef., DualRef-L, DualRef-S), respectively. In all output BAM files from each tool, samtools was used to extract the read IDs of all included reads. From the read IDs extracted from the output BAM file, the read IDs of the human sample and the read IDs of mouse sample are counted separately. Based on this count, the TPR, FPR, sensitivity, specificity, precision, accuracy, and *F*-score of each tool are calculated. All TPR and FPR values were calculated from the mean values of three random seed replicates. Next, all the result files of each filtering methods are compared with the file before filtering to obtain the reduced *H*_*f*_ of all HAMA position. By summing all reduced *H*_*f*_ values, the total sum of reduced *H*_*f*_ value was obtained. Callable HAMAs are collected from the result files of GATK4 CollectAllelicCounts (ver. 4.1.1.0). First, all mismatched bases were extracted, and all bases with a VAF value of 0.5 or less and an ALT count of 5 or less were removed.

### Evaluation of HAMA blacklisting

First, the filtering methods are applied to all benchmark datasets, and each BAM file is divided into human-derived reads and mouse-derived reads. Next, all mismatch bases were extracted by using GATK4 CollectAllelicCounts (ver. 4.1.1.0) for the divided BAM files. A human-derived somatic variant and a mouse-derived somatic variant were defined by comparing the separately obtained mismatch bases with somatic variant call results using GATK4 Mutect2 (ver. 4.1.1.0). The number of HAMA blacklist applied to mouse-derived somatic variant was counted as TP, and the number of HAMA blacklist applied to human-derived somatic variant was counted as FP.

## Supplementary information

**Additional file 1.** Supplementary figures and tables.

**Additional file 2.** Read counts, RPKMs of mouse reads captured with SureSelectHumanAllExon V5.

**Additional file 3.** GO annotation analysis. GO annotation analysis of mouse reads aligned on human genome reference.

**Additional file 4.** HAMA list. List of all HAMA found in this study.

**Additional file 5.** Review history.

## Data Availability

The WES data of human lung cancer (TCGA-67-3771-01A-01D, TCGA-73-4658-01A-01D) are available at TCGA (https://portal.gdc.cancer.gov/) [44]. The WES data of five normal mice DNA captured with human WES kit are available at SRA, under accession code PRJNA545013 [45]. The gnomAD mean coverage data is available at gnomAD (https://gnomad.broadinstitute.org/) [26]. The scripts used in this study including contamination estimation code are available on GitHub [46] and Zenodo [47].
