# Supplementary material for: Impact of mouse contamination in genomic profiling of patient-derived models and best practice for robust analysis
Source: Genome Biol. 2019 Nov 11;20:231. doi: 10.1186/s13059-019-1849-2 (PMC6844030; doi:10.1186/s13059-019-1849-2)
Supplement: Supplementary file 1 — Additional file 1. Supplementary figures and tables. [file 13059_2019_1849_MOESM1_ESM.docx]

**Additional file 1: Supplementary Information**

Impact of mouse contamination in genomic profiling of
patient-derived models and best practice for robust analysis

Se-Young Jo^1,†^, Eunyoung Kim^1,†^, and Sangwoo Kim^1,*^

^1^Department of Biomedical Systems Informatics and Brain Korea 21 PLUS Project for Medical Science, Yonsei University College of Medicine, Seoul 03722, South Korea

^†^These authors contributed equally

^*^To whom the correspondence should be addressed: [swkim@yuhs.ac](mailto:swkim@yuhs.ac)

### **Contents**

[Supplementary Methods 3](#_Toc20754472)

[**Calculation of RPKM of mouse-reads on human genome reference** 3](#_Toc20754473)

[**GO annotation analysis** 3](#_Toc20754474)

[**Actual command line for performing filtering tools** 3](#_Toc20754475)

[Supplementary Figures 6](#_Toc20754476)

[**Figure S1** Read count and RPKM of mouse-reads on human genome reference 6](#_Toc20754477)

[**Figure S2** Top Ranked Genes in RPKM values 7](#_Toc20754478)

[**Figure S3** Median *H_f_* values from five global contamination levels. 8](#_Toc20754479)

[**Figure S4** Percentage of COSMIC variants that coincide with HAMA 9](#_Toc20754480)

[**Figure S5** Flowchart of mouse read filtering methods 10](#_Toc20754481)

[**Figure S6** *H_f_* (HAMA allele frequency) distribution changes after filtering 11](#_Toc20754482)

[**Figure S7** The efficiency of strict and lenient blacklisting 12](#_Toc20754483)

[**Figure S8** The efficiency of using strain-specific references 13](#_Toc20754484)

[Supplementary Tables 14](#_Toc20754485)

[**Table S1** *in silico* prepared benchmark dataset 14](#_Toc20754486)

[**Table S2** Strain specificity of 3 mouse strains on human genome reference 15](#_Toc20754487)

[**Table S3** The result of HAMA blacklisting by contamination ratio and filtering method 16](#_Toc20754488)

[**Table S4** A summary of the system requirements and speeds of filtering methods 17](#_Toc20754489)

# **Supplementary Methods**

## **Calculation of RPKM of mouse-reads on human genome reference**

A BED file contains all targeted area of SureSelect Human All Axon V5 has been obtained from SureDesign web page. (<https://earray.chem.agilent.com/suredesign/>) Then counted all the reads aligned to each region using GATK CollectReadCounts. RPKM (reads per kilobase per million mapped reads) has been calculated with following formula:

$$RPKM= \frac{Number of reads of the region}{Total reads per Mb \times Region length per Kb}$$

## **GO annotation analysis**

GO annotation analysis is performed with 5,917 gene sets in the Molecular Signatures Database (ver. 6.2 MSigDB). The GO terms in Gene Ontotogy(GO) annotation sets (C5 collection) are consist of three GO ontologies: molecular function (MF), cellular component (CC) and biological process (BP). The ranking of the average RPKM values were used to infer biological functions related with mouse-reads aligned on human genome reference.

## **Actual command line for performing filtering tools**

***BBsplit***

/BBMap/bbsplit.sh **\**

build=1 **\**

ref_Mouse=mm10.fa **\**

ref_Human=hg19.fa

/BBMap/bbsplit.sh **\**

build=1 **\**

in=*${*SAMPLE*}*_1.fq **\**

in2=*${*SAMPLE*}*_2.fq **\**

basename=*${*OutputPath*}*/*${*SAMPLE*}* _%_#.fq

***Xenome***

xenome index **\**

-G hg19.fa **\**

-H mm10.fa **\**

-P *${*IDXPath*}*/idx

xenome classify **\**

-P *${*IDXPath*}*/idx

--pairs **\**

--graft-name human **\**

--host-name mouse **\**

--output-filename-prefix *${*OutputPath*}*/*${*SAMPLE*}* **\**

-i *${*DataPath*}${*SAMPLE*}*_1.fq **\**

-i *${*DataPath*}${*SAMPLE*}*_2.fq

***Bamcmp***

/bamcmp-master/build/bamcmp -n **\**

-1 *${*SAMPLE*}*.aligned_to_hg19.bam **\**

-2 *${*SAMPLE*}*.aligned_to_mm10.bam **\**

-a *${*OutputPath*}*/*${*SAMPLE*}*.bamcmp.humanOnly.bam **\**

-A *${*OutputPath*}*/*${*SAMPLE*}*.bamcmp.humanBetter.bam **\**

-s match

samtools merge *${*OutputPath*}*/*${*SAMPLE*}*.bamcmp.bam **\**

*${*OutputPath*}*/*${*SAMPLE*}*.bamcmp.humanOnly.bam **\**

*${*OutputPath*}*/*${*SAMPLE*}*.bamcmp.humanBetter.bam

***Disambiguate***

python /disambiguate/disambiguate.py **\**

-o *${*OutputPath*}*/ **\**

-d **\**

-a bwa **\**

*${*SAMPLE*}*.aligned_to_hg19.bam **\**

*${*SAMPLE*}*.aligned_to_mm10.bam

***XenofilteR***

library("XenofilteR")

bp.param <- SnowParam(workers=1, type = "SOCK")

input.list <- c(${SAMPLE} .aligned_to_hg19.bam, ${SAMPLE} .aligned_to_mm10.bam)

XenofilteR(input.list, destination.folder = "/${OutputPath}/", bp.param = bp.param)

***ConcatRef***

*# 1. Extract hg19 aligned reads*

samtools view -b *${*SAMPLE*}*.aligned_to_ConcatRef.bam chrM.hg19 chr1.hg19 … chrX.hg19 chrY.hg19 > *${*SAMPLE*}*.aligned_to_ConcatRef.hg19_only.bam

*# 2. Replace BAM header*

samtools view -H *${*SAMPLE*}*.aligned_to_ConcatRef.hg19_only.bam | sed -e 's/SN:\(chr[0-9MXY]\).hg19/SN:\1/' -e 's/SN:\(chr[0-9][0-9]\).hg19/SN:\1/' | samtools reheader - *${*SAMPLE*}*.aligned_to_ConcatRef.hg19_only.bam > *${*SAMPLE*}*.aligned_to_ConcatRef.hg19_only.reheadered.bam

*# 3. Remove remained RNEXT mm10 reads*

samtools view -h *${*SAMPLE*}*.aligned_to_ConcatRef.hg19_only.reheadered.bam | awk -F '\t' '{ if (index($7,"mm10")==0) print $0 }' | samtools view -bS > *${*SAMPLE*}*.ConcatRef.bam

***DualRef-L***

samtools view -F 4 *${*SAMPLE*}*.aligned_to_mm10.bam | awk $0 ~"NM:i:0" | cut -f1 | **\**

sort -u > *${*SAMPLE*}*.NM0.readID.txt

gatk FilterSamReads **\**

--INPUT *${*SAMPLE*}*.aligned_to_hg19.bam **\**

--OUTPUT *${*SAMPLE*}*.aligned_to_hg19.filtered.bam **\**

--READ_LIST_FILE *${*SAMPLE*}*.NM0.readID.txt **\**

--FILTER excludeReadList **\**

--CREATE_INDEX true

***DualRef-S***

samtools view -F 4 *${*SAMPLE*}*.aligned_to_mm10.bam | cut -f1 | **\**

sort -u > *${*SAMPLE*}*.readID.txt

gatk FilterSamReads **\**

--INPUT *${*SAMPLE*}*.aligned_to_hg19.bam **\**

--OUTPUT *${*SAMPLE*}*.aligned_to_hg19.filtered.bam **\**

--READ_LIST_FILE *${*SAMPLE*}*.readID.txt **\**

--FILTER excludeReadList **\**

--CREATE_INDEX true

# **Supplementary Figures**

## **Figure S1** Read count and RPKM of mouse-reads on human genome reference

(a) Read count of all targeted regions has been obtained through GATK4 CollectReadCount. Every region has been annotated and merged with NCBI RefSeq gene name. (b) These gene count has been normalized with RPKM calculation.

## **Figure S2** Top Ranked Genes in RPKM values

(a) Top ranked read count per gene has been shown. Left graph shows total gene provide by RefSeq gene list. The red bar indicates a gene belonging to CGC, which is a cancer related gene list published by the Sanger Institute. (b) In the same manner of (a), top ranked RPKM has been shown.

## **Figure S3** Median *H_f_* values from five global contamination levels.

Simple linear regression shows their linear correlation and the estimated slope, which converts an observed median *H_f_* value of a sample to its global contamination level (and *vice versa*). The results from the hypothetical contaminated dataset generated in this experiment showed a very significant linear correlation up to 50%, and tended to deviate from linearity in 80% of extreme contaminated condition.

## **Figure S4** Percentage of COSMIC variants that coincide with HAMA

In descending order of the percentage of variants that coincide with HAMA for all sample origin registered in the COSMIC v88 database. Of the total 4,843,731 COSMIC variants, 2.11% were consistent with HAMA, and all sample origins showed the same or smaller HAMA proportion except short-term culture and organoid culture. These two sample origins showed significantly high proportion of HAMA variants, suggesting that the false variants derived from mouse genome were registered with high probability, rather than coincide by chance.

## **Figure S5** Flowchart of mouse read filtering methods

(a) Pre-alignment filtering tools (b) Post-alignment filtering tools (c) ConcatRef (d) DualRef-S (e) DualRef-L


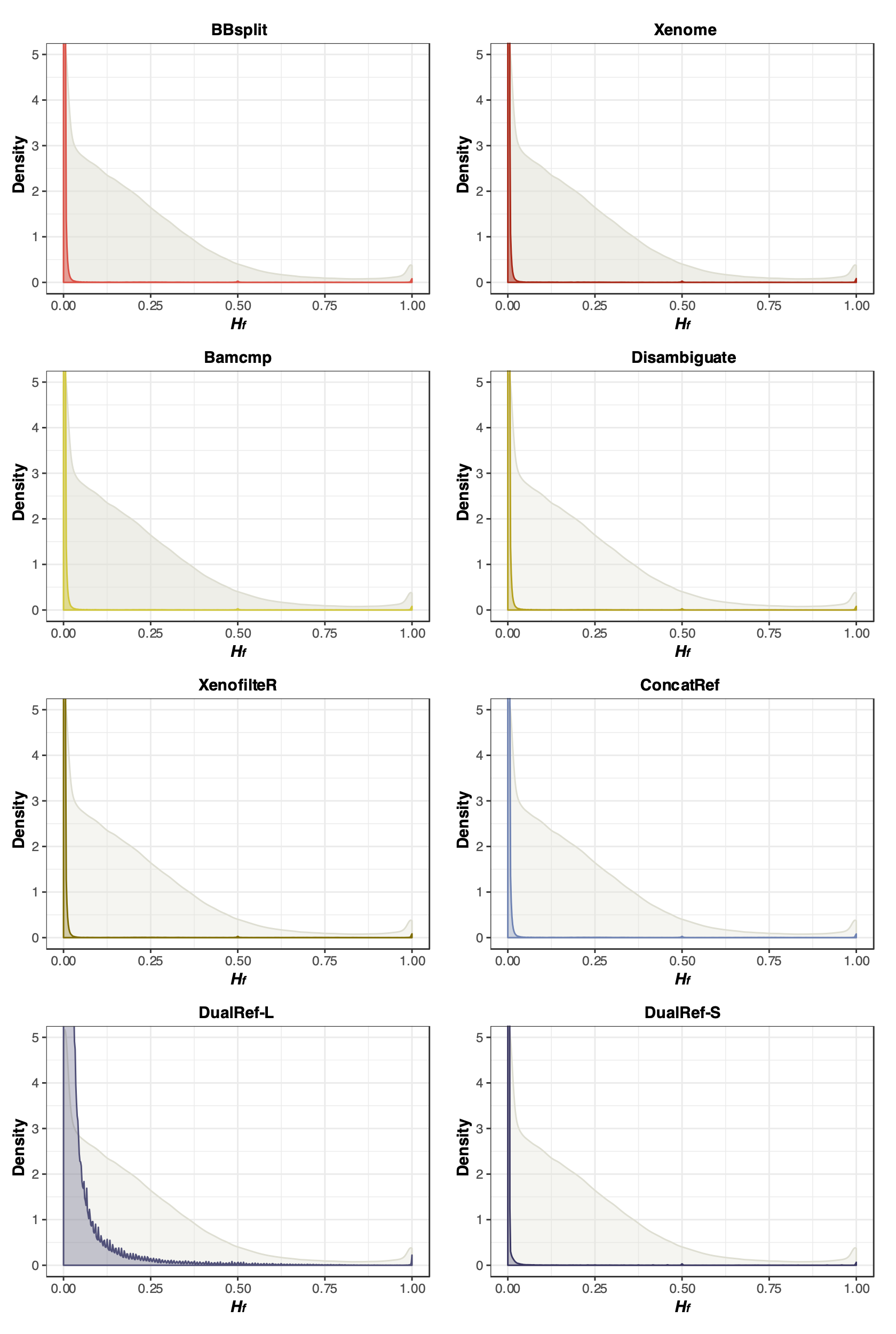


## **Figure S6** *H_f_* (HAMA allele frequency) distribution changes after filtering

*Hf* distribution of unfiltered dataset has been show with light grey histogram on every plot. The *Hf* distributions of each tool’s filtering result files have been rendered with histogram together.

## **Figure S7** The efficiency of strict and lenient blacklisting

Before and after applying further filtering with HAMAlist to the somatic variation calling has been rendered. All of the filtering method performed well and the Mutect2 (GATK ver. 4.1.1.0) called less than five false variants derived from mouse. However, applying HAMA blacklist to this result file, could filter out more false variant (< 2).

Lenient blacklisting (high-risk HAMA) can filter out almost equal amount of false variant with less loss of true variants.

## **Figure S8** The efficiency of using strain-specific references

Using strain-specific references in the application of the mouse read filtering methods did not show any benefit.

# **Supplementary Tables**

| No. | Human | Mouse | Contam. Ratio | Random Seed | No. | Human | Mouse | Contam. Ratio | Random Seed |
| --- | --- | --- | --- | --- | --- | --- | --- | --- | --- |
| 1 | TCGA-67 | A/J no.1 | 5% | 10 | **76** | TCGA-73 | A/J no.1 | 5% | 10 |
| 2 | TCGA-67 | A/J no.1 | 5% | 20 | **77** | TCGA-73 | A/J no.1 | 5% | 20 |
| 3 | TCGA-67 | A/J no.1 | 5% | 30 | **78** | TCGA-73 | A/J no.1 | 5% | 30 |
| 4 | TCGA-67 | A/J no.1 | 10% | 10 | **79** | TCGA-73 | A/J no.1 | 10% | 10 |
| 5 | TCGA-67 | A/J no.1 | 10% | 20 | **80** | TCGA-73 | A/J no.1 | 10% | 20 |
| 6 | TCGA-67 | A/J no.1 | 10% | 30 | **81** | TCGA-73 | A/J no.1 | 10% | 30 |
| 7 | TCGA-67 | A/J no.1 | 20% | 10 | **82** | TCGA-73 | A/J no.1 | 20% | 10 |
| 8 | TCGA-67 | A/J no.1 | 20% | 20 | **83** | TCGA-73 | A/J no.1 | 20% | 20 |
| 9 | TCGA-67 | A/J no.1 | 20% | 30 | **84** | TCGA-73 | A/J no.1 | 20% | 30 |
| 10 | TCGA-67 | A/J no.1 | 50% | 10 | **85** | TCGA-73 | A/J no.1 | 50% | 10 |
| 11 | TCGA-67 | A/J no.1 | 50% | 20 | **86** | TCGA-73 | A/J no.1 | 50% | 20 |
| 12 | TCGA-67 | A/J no.1 | 50% | 30 | **87** | TCGA-73 | A/J no.1 | 50% | 30 |
| 13 | TCGA-67 | A/J no.1 | 80% | 10 | **88** | TCGA-73 | A/J no.1 | 80% | 10 |
| 14 | TCGA-67 | A/J no.1 | 80% | 20 | **89** | TCGA-73 | A/J no.1 | 80% | 20 |
| 15 | TCGA-67 | A/J no.1 | 80% | 30 | **90** | TCGA-73 | A/J no.1 | 80% | 30 |
| 16 | TCGA-67 | A/J no.2 | 5% | 10 | **91** | TCGA-73 | A/J no.2 | 5% | 10 |
| 17 | TCGA-67 | A/J no.2 | 5% | 20 | **92** | TCGA-73 | A/J no.2 | 5% | 20 |
| 18 | TCGA-67 | A/J no.2 | 5% | 30 | **93** | TCGA-73 | A/J no.2 | 5% | 30 |
| 19 | TCGA-67 | A/J no.2 | 10% | 10 | **94** | TCGA-73 | A/J no.2 | 10% | 10 |
| 20 | TCGA-67 | A/J no.2 | 10% | 20 | **95** | TCGA-73 | A/J no.2 | 10% | 20 |
| 21 | TCGA-67 | A/J no.2 | 10% | 30 | **96** | TCGA-73 | A/J no.2 | 10% | 30 |
| 22 | TCGA-67 | A/J no.2 | 20% | 10 | **97** | TCGA-73 | A/J no.2 | 20% | 10 |
| 23 | TCGA-67 | A/J no.2 | 20% | 20 | **98** | TCGA-73 | A/J no.2 | 20% | 20 |
| 24 | TCGA-67 | A/J no.2 | 20% | 30 | **99** | TCGA-73 | A/J no.2 | 20% | 30 |
| 25 | TCGA-67 | A/J no.2 | 50% | 10 | **100** | TCGA-73 | A/J no.2 | 50% | 10 |
| 26 | TCGA-67 | A/J no.2 | 50% | 20 | **101** | TCGA-73 | A/J no.2 | 50% | 20 |
| 27 | TCGA-67 | A/J no.2 | 50% | 30 | **102** | TCGA-73 | A/J no.2 | 50% | 30 |
| 28 | TCGA-67 | A/J no.2 | 80% | 10 | **103** | TCGA-73 | A/J no.2 | 80% | 10 |
| 29 | TCGA-67 | A/J no.2 | 80% | 20 | **104** | TCGA-73 | A/J no.2 | 80% | 20 |
| 30 | TCGA-67 | A/J no.2 | 80% | 30 | **105** | TCGA-73 | A/J no.2 | 80% | 30 |
| 31 | TCGA-67 | BALB/c no.1 | 5% | 10 | **106** | TCGA-73 | BALB/c no.1 | 5% | 10 |
| 32 | TCGA-67 | BALB/c no.1 | 5% | 20 | **107** | TCGA-73 | BALB/c no.1 | 5% | 20 |
| 33 | TCGA-67 | BALB/c no.1 | 5% | 30 | **108** | TCGA-73 | BALB/c no.1 | 5% | 30 |
| 34 | TCGA-67 | BALB/c no.1 | 10% | 10 | **109** | TCGA-73 | BALB/c no.1 | 10% | 10 |
| 35 | TCGA-67 | BALB/c no.1 | 10% | 20 | **110** | TCGA-73 | BALB/c no.1 | 10% | 20 |
| 36 | TCGA-67 | BALB/c no.1 | 10% | 30 | **111** | TCGA-73 | BALB/c no.1 | 10% | 30 |
| 37 | TCGA-67 | BALB/c no.1 | 20% | 10 | **112** | TCGA-73 | BALB/c no.1 | 20% | 10 |
| 38 | TCGA-67 | BALB/c no.1 | 20% | 20 | **113** | TCGA-73 | BALB/c no.1 | 20% | 20 |
| 39 | TCGA-67 | BALB/c no.1 | 20% | 30 | **114** | TCGA-73 | BALB/c no.1 | 20% | 30 |
| 40 | TCGA-67 | BALB/c no.1 | 50% | 10 | **115** | TCGA-73 | BALB/c no.1 | 50% | 10 |
| 41 | TCGA-67 | BALB/c no.1 | 50% | 20 | **116** | TCGA-73 | BALB/c no.1 | 50% | 20 |
| 42 | TCGA-67 | BALB/c no.1 | 50% | 30 | **117** | TCGA-73 | BALB/c no.1 | 50% | 30 |
| 43 | TCGA-67 | BALB/c no.1 | 80% | 10 | **118** | TCGA-73 | BALB/c no.1 | 80% | 10 |
| 44 | TCGA-67 | BALB/c no.1 | 80% | 20 | **119** | TCGA-73 | BALB/c no.1 | 80% | 20 |
| 45 | TCGA-67 | BALB/c no.1 | 80% | 30 | **120** | TCGA-73 | BALB/c no.1 | 80% | 30 |
| 46 | TCGA-67 | C57BL/6 no.1 | 5% | 10 | **121** | TCGA-73 | C57BL/6 no.1 | 5% | 10 |
| 47 | TCGA-67 | C57BL/6 no.1 | 5% | 20 | **122** | TCGA-73 | C57BL/6 no.1 | 5% | 20 |
| 48 | TCGA-67 | C57BL/6 no.1 | 5% | 30 | **123** | TCGA-73 | C57BL/6 no.1 | 5% | 30 |
| 49 | TCGA-67 | C57BL/6 no.1 | 10% | 10 | **124** | TCGA-73 | C57BL/6 no.1 | 10% | 10 |
| 50 | TCGA-67 | C57BL/6 no.1 | 10% | 20 | **125** | TCGA-73 | C57BL/6 no.1 | 10% | 20 |
| 51 | TCGA-67 | C57BL/6 no.1 | 10% | 30 | **126** | TCGA-73 | C57BL/6 no.1 | 10% | 30 |
| 52 | TCGA-67 | C57BL/6 no.1 | 20% | 10 | **127** | TCGA-73 | C57BL/6 no.1 | 20% | 10 |
| 53 | TCGA-67 | C57BL/6 no.1 | 20% | 20 | **128** | TCGA-73 | C57BL/6 no.1 | 20% | 20 |
| 54 | TCGA-67 | C57BL/6 no.1 | 20% | 30 | **129** | TCGA-73 | C57BL/6 no.1 | 20% | 30 |
| 55 | TCGA-67 | C57BL/6 no.1 | 50% | 10 | **130** | TCGA-73 | C57BL/6 no.1 | 50% | 10 |
| 56 | TCGA-67 | C57BL/6 no.1 | 50% | 20 | **131** | TCGA-73 | C57BL/6 no.1 | 50% | 20 |
| 57 | TCGA-67 | C57BL/6 no.1 | 50% | 30 | **132** | TCGA-73 | C57BL/6 no.1 | 50% | 30 |
| 58 | TCGA-67 | C57BL/6 no.1 | 80% | 10 | **133** | TCGA-73 | C57BL/6 no.1 | 80% | 10 |
| 59 | TCGA-67 | C57BL/6 no.1 | 80% | 20 | **134** | TCGA-73 | C57BL/6 no.1 | 80% | 20 |
| 60 | TCGA-67 | C57BL/6 no.1 | 80% | 30 | **135** | TCGA-73 | C57BL/6 no.1 | 80% | 30 |
| 61 | TCGA-67 | C57BL/6 no.2 | 5% | 10 | **136** | TCGA-73 | C57BL/6 no.2 | 5% | 10 |
| 62 | TCGA-67 | C57BL/6 no.2 | 5% | 20 | **137** | TCGA-73 | C57BL/6 no.2 | 5% | 20 |
| 63 | TCGA-67 | C57BL/6 no.2 | 5% | 30 | **138** | TCGA-73 | C57BL/6 no.2 | 5% | 30 |
| 64 | TCGA-67 | C57BL/6 no.2 | 10% | 10 | **139** | TCGA-73 | C57BL/6 no.2 | 10% | 10 |
| 65 | TCGA-67 | C57BL/6 no.2 | 10% | 20 | **140** | TCGA-73 | C57BL/6 no.2 | 10% | 20 |
| 66 | TCGA-67 | C57BL/6 no.2 | 10% | 30 | **141** | TCGA-73 | C57BL/6 no.2 | 10% | 30 |
| 67 | TCGA-67 | C57BL/6 no.2 | 20% | 10 | **142** | TCGA-73 | C57BL/6 no.2 | 20% | 10 |
| 68 | TCGA-67 | C57BL/6 no.2 | 20% | 20 | **143** | TCGA-73 | C57BL/6 no.2 | 20% | 20 |
| 69 | TCGA-67 | C57BL/6 no.2 | 20% | 30 | **144** | TCGA-73 | C57BL/6 no.2 | 20% | 30 |
| 70 | TCGA-67 | C57BL/6 no.2 | 50% | 10 | **145** | TCGA-73 | C57BL/6 no.2 | 50% | 10 |
| 71 | TCGA-67 | C57BL/6 no.2 | 50% | 20 | **146** | TCGA-73 | C57BL/6 no.2 | 50% | 20 |
| 72 | TCGA-67 | C57BL/6 no.2 | 50% | 30 | **147** | TCGA-73 | C57BL/6 no.2 | 50% | 30 |
| 73 | TCGA-67 | C57BL/6 no.2 | 80% | 10 | **148** | TCGA-73 | C57BL/6 no.2 | 80% | 10 |
| 74 | TCGA-67 | C57BL/6 no.2 | 80% | 20 | **149** | TCGA-73 | C57BL/6 no.2 | 80% | 20 |
| 75 | TCGA-67 | C57BL/6 no.2 | 80% | 30 | **150** | TCGA-73 | C57BL/6 no.2 | 80% | 30 |

##

## **Table S1** *in silico* prepared benchmark dataset

| Description | Symbol | Numer of HAMA |
| --- | --- | --- |
| A/J no.1 | M1 | **1,466,852** |
| A/J no.2 | M2 | **1,491,949** |
| BALB/c no.1 | M3 | **1,491,563** |
| C57BL/6 no.1 | M4 | **1,495,793** |
| C57BL/6 no.2 | M5 | **1,490,144** |
| Union of all mouse | M1 ∪ M2 ∪ M3 ∪ M4 ∪ M5 | **1,800,077** |
| Intersection of all mouse | M1 ∩ M2 ∩ M3 ∩ M4 ∩ M5 | **1,602,035** |
| A/J common HAMA | (M1 ∩ M2) | **1,344,262** |
| BALB/c common HAMA | M3 | **1,491,563** |
| C57BL/6 common HAMA | (M4 ∩ M5) | **1,362,658** |
| Union of 3 strains | (M1 ∩ M2) ∪ M3 ∪ (M4 ∩ M5) | **1,602,035** |
| Intersection of 3 strains | (M1 ∩ M2) ∩ M3 ∩ (M4 ∩ M5) | **1,207,556** |
| A/J specific HAMA | (M1 ∩ M2) – M3 – M4 – M5 | **35,137** |
| BALB/c specific HAMA | M3 – (M1 ∩ M2) – (M4 ∩ M5) | **41,435** |
| C57BL/6 specific HAMA | (M4 ∩ M5) – (M1 ∩ M2) – M3 | **46,229** |
| A/J & BALB/c common HAMA | (M1 ∩ M2) ∩ M3 | **72,463** |
| BALB/c & C57BL/6 common HAMA | M3 ∩ (M4 ∩ M5) | **79,767** |
| C57BL/6 & A/J common HAMA | (M4 ∩ M5) ∩ (M1 ∩ M2) | **29,106** |

## **Table S2** Strain specificity of 3 mouse strains on human genome reference

| **Contamination Ratio** | **Human-derived variants** | **Lost human-derived variants with** | | **Mouse-derived variants** | **Filtered mouse-derived variants with** | |
| --- | --- | --- | --- | --- | --- | --- |
|  |  | **Lenient Blacklisting** | **Strict**  **Blacklisting** |  | **Lenient Blacklisting** | **Strict**  **Blacklisting** |
| **5%** | 1312.0 | 5.5 | 15.2 | 2.3 | 1.5 | 1.5 |
| **10%** | 1257.7 | 5.4 | 14.2 | 3.2 | 2.0 | 2.0 |
| **20%** | 1223.8 | 5.0 | 13.4 | 3.1 | 2.2 | 2.3 |
| **50%** | 1165.4 | 5.2 | 12.9 | 3.4 | 2.5 | 2.8 |
| **Filtering Methods** | **Human-derived variants** | **Lost human-derived variants with** | | **Mouse-derived variants** | **Filtered mouse-derived variants with** | |
|  |  | **Lenient Blacklisting** | **Strict**  **Blacklisting** |  | **Lenient Blacklisting** | **Strict**  **Blacklisting** |
| **BBsplit** | 1274.0 | 5.9 | 15.2 | 3.2 | 2.4 | 2.7 |
| **Bamcmp** | 1273.6 | 6.3 | 15.7 | 4.6 | 2.8 | 2.9 |
| **ConcatRef** | 1272.7 | 5.1 | 14.5 | 2.7 | 1.7 | 1.8 |
| **Disambiguate** | 1274.5 | 6.4 | 15.7 | 4.1 | 2.7 | 2.7 |
| **DualRef-S** | 864.2 | 1.1 | 4.8 | 1.6 | 1.6 | 1.6 |
| **XenofilteR** | 1262.7 | 5.7 | 14.2 | 2.0 | 1.6 | 1.6 |
| **Xenome** | 1263.2 | 3.2 | 10.3 | 2.0 | 1.7 | 1.7 |

## **Table S3** The result of HAMA blacklisting by contamination ratio and filtering method

The numbers of somatic variant calls before and after applying HAMA blacklist. All numbers are mean value of entire dataset filtered by all filtering methods except DualRef-L. In terms of filtering ability of mouse-derived variants, HAMA and high-risk HAMA blacklisting were equally effective. Therefore, it is recommended to apply lenient blacklisting (high-risk HAMA blacklisting) to reduce false positive (loss of human-derived variants).

| Tools | Input file type | Operating System | Memory Requirement | Programming Language | Library Dependencies | Running Time^*^ |
| --- | --- | --- | --- | --- | --- | --- |
| BBsplit | FASTQ | Linux, MAC, Windows | 32G | JAVA | - | ~ 6 hours |
| Xenome | FASTQ | Linux | 32G | C++ | g++, cmake, libboost-all-dev, pandoc, zlib1g-dev, libbz2-dev, libsqlite3-dev | ~ 72 hours |
| Bamcmp | SAM/BAM | Linux | 4G | C++ | htslib | ~ 2 hours |
| Disambiguate | SAM/BAM | Linux | 4G | C++, Python | zlib, Bamtools C++ API / Pysam | ~ 5 hours |
| XenofilteR | SAM/BAM | Linux, Mac, Windows | 32G | R | Rsamtools, GenomicAlignments, BiocParallel, futile.logger | ~ 1 hours |
| ConcatRef | SAM/BAM | Linux, Mac | 4G | Bash | - | ~ 1 hour |
| DualRef | SAM/BAM | Linux, Mac | 4G | Bash | - | ~ 1 hour |

## **Table S4** A summary of the system requirements and speeds of filtering methods

*Please note that the running time is measured only the actual filtering step of each method. The time required to prepare the input for each method can vary.
