# Supplementary material for: Impact of mouse contamination in genomic profiling of patient-derived models and best practice for robust analysis
Source: Genome Biol. 2019 Nov 11;20:231. doi: 10.1186/s13059-019-1849-2 (PMC6844030; doi:10.1186/s13059-019-1849-2)
Supplement: Supplementary file 5 — Additional file 5. Review history. [file 13059_2019_1849_MOESM5_ESM.docx]

Review History

**First round of review**

**Reviewer 1**

**Comments to author:**

The in vivo PDX and in vitro cell-line models are important technologies in clinical cancer research as the development of the cancer cells in these agents hold similarity to the original ones in patients which provides meaningful insights and treatment tests. However, the mouse genome mixed into the sequencing results of these technologies jeopardize the downstream analyses. Although several filtering methods have been proposed to address these issues, there is still a demand for good ground truth benchmark dataset which could be adopted to measure the effectiveness of the filtration methods, and to demonstrate the exact impact of the mouse genome in genomic analysis. The authors constructed a benchmark dataset of 5 liver tissues from 3 mouse strains using human whole exome sequencing kit. Analyses indicated 38.7% alleles with high sensitivity to contamination, which are pervasive causes of false cancer mutations in public databases and are signatures for predicting global contamination. The authors then evaluated 8 filtering methods in terms of mouse-read filtration and reduction of mouse-specific alleles. They found that all filtering tools generally performed well with differences in strictness of algorithm and efficiency of mouse allele removal were observed. The authors suggested a best practice pipeline that contains the estimation of contamination level, mouse-read filtration and variant filtration. The authors finally claimed that the inclusion of mouse cells in patient-derived models hinders genomic analysis and should be addressed carefully and the suggested guidelines improve the robustness and maximize the utility of genomic analysis of these models.

This work is an important patch in cancer research and cancer therapy, especially in the scenario where the downstream application is for conducting personalized treatment to patients. Here are a few issues that the authors need to address and/or discuss before being considered for publication.

Major issues:

1.     In the Background section, the statements that discuss the motivation of this study need more references to support. For example, in Page 2 line 40-42, the authors stated “Indeed, research has shown that resected PDX samples can harbor up to 80% murine DNA without a mistake in the separation process”, more references are needed here and what situation could 80% of murine DNA be mixed into the sequencing library need to be discussed? Same problem applies to the following in vitro statements; and in the last sentence, “Accordingly, researchers have lobbied continuing demands for efficient tools with which to deconvolute or remove murine effects in genomic analyses of PDM models”, again reference.

2.     When conducting the analyses and statistics, read length is set to 100-bp, but according to the current NGS technology, usually 1-10 bp and bp after 60 or 100 contain low quality readout, therefore adding another level of noise to the human-mouse mixture model. The authors need to experiment different read length to justify their findings. This applies to both DNA-seq and RNA-seq, as the former holds the false SNV/SNP calling and the latter trades off the reads mappability with accuracy.


3.     As the author stated that up to 80% of murine DNA could be mixed into the human reads, then why the noise level are only tested on up to 50%? 70% 80%, and even 90% noise level need to be tested as well to include extreme cases.

4.     Specifically, how could an end user estimate the contamination level need to be discussed in detail?

5.     In cancer genome, the occurrence of somatic SNV is not totally random but somehow shaped by its 3D genome environment (refer to the Somatic Co-mutation Hotspot (SCH) theory in https://www.nature.com/articles/srep35270). How could this kind of prior knowledge affect or help the filtration step need to be discussed.

6.     Essentially, the wet lab tracer technology that might be able to provide single-cell level cell origin information (either murine or human cancer) is the game changer for this sub-area. The current advances for this part need to be included in the Discussion section of this work.


Minor issues:
1.     The BWA parameters are set default? What is the default? And how allowing different levels of gap and mismatch could affect the analyses need to be discussed.
2.     The color scheme in Figure 5D is not very distinguishing, richer contract color scheme is advised.
3.     The formulas in this manuscript need to be presented in a more mathematical way rather than descriptive.

**Reviewer 2**

**Comments to author:**

The authors of the study attempt to address the important problem of removing mouse reads that often contaminate human reads in studies involving patient-derived xenografts. Since mouse DNA fragments could map quite comfortably to highly conserved regions in the human genome, failing to remove mouse DNA could result in false calls and false interpretations by mutations detection methods. This makes genetic analysis of patient derived xenografts more tedious than original tumor samples. Here, the authors benchmark eight tools that filter mouse reads using simulated datasets with specific mouse contamination levels and subsequently provide best practices for future studies.

Here are some of the comments about the paper,
The authors describe the results using human lung cancer cells from TCGA consortium. They use reads from mouse liver to simulate the contamination. However, they do not discuss their reasoning behind the choices for these specific cell types. Given the tremendous heterogeneity associated with cancer cells, the authors should discuss if their results would generalize to all types of cancer cells, regardless of the tissue. Since the authors ran multiple tools to benchmark them, they should comment on the computational speed and requirements for the tools they compared. Their experimental design to simulate data by mixing mouse reads with human reads with mouse cells amounting to 5, 10, 20 or 50% of the total reads with three replicates is robust. Overall a good paper.

**Reviewer 3**

**Comments to author:**

Patient-derived models (PDM) are popular in clinical cancer research. The inevitable inclusion of mouse genome in patient-derived models is a remaining concern in genomic analysis due to the genomic similarity in coding regions between humans and mice. Multiple tools and strategies have been developed for mouse-read filtration. In this manuscript, the authors constructed a benchmark to demonstrate the impact of mouse reads in genomic analysis and to assess the performance of eight filtering methods in terms of mouse-read filtration and reduction of mouse-specific alleles. Furthermore, the authors gave a practice pipeline for variant analysis when analyzing human genome data contaminated by mouse genome. Overall, this is some useful benchmark study. I have several comments as described below.

1.     Although the results in Fig. S6 showed that the average of false variants by “filtering method only” was higher than the average of false variants by “filtering method plus Lenient Blacklist” or by “filtering method plus Strict Blacklist” (2.9 VS 0.8 or 2.9 VS 0.7), the average of loss of true variants by “filtering method only” was lower than the average of loss of true variants by “filtering method plus Lenient Blacklist” or by “filtering method plus Strict Blacklist” (0 VS 4.8 or 0 VS 11.5). Therefore, it is hard to claim “filtering method plus Lenient Blacklist” or “filtering method plus Strict Blacklist” performs better than “filtering method only in calling variants”. 
Furthermore, given the small numbers (false variants and true variants), the existing methods (filtering method only) seem working very well. Do we really need the two suggested pipelines?
2.     Is the estimate of global contamination level reliable?  1) the four H_f data points in Fig 2F are means of repeated samples. How fluctuating are those H_f from a single sample?  In realistic experiments, you have only one sample. 
3.     In page 8 line 57, “(Table 1, see Methods for detailed benchmark procedures).”Table 1 is missing.
4.     In Fig. S6, the x-labels, “Filtering method + Lenient Blacklisting” and “Filtering method + Strict Blacklisting” are misplaced.

**Authors Response**

**Point-by-point responses to the reviewers’ comments:**

Reviewer #1: The in vivo PDX and in vitro cell-line models are important technologies in clinical cancer research as the development of the cancer cells in these agents hold similarity to the original ones in patients which provides meaningful insights and treatment tests. However, the mouse genome mixed into the sequencing results of these technologies jeopardize the downstream analyses. Although several filtering methods have been proposed to address these issues, there is still a demand for good ground truth benchmark dataset which could be adopted to measure the effectiveness of the filtration methods, and to demonstrate the exact impact of the mouse genome in genomic analysis. The authors constructed a benchmark dataset of 5 liver tissues from 3 mouse strains using human whole exome sequencing kit. Analyses indicated 38.7% alleles with high sensitivity to contamination, which are pervasive causes of false cancer mutations in public databases and are signatures for
predicting global contamination. The authors then evaluated 8 filtering methods in terms of mouse-read filtration and reduction of mouse-specific alleles. They found that all filtering tools generally performed well with differences in strictness of algorithm and efficiency of mouse allele removal were observed. The authors suggested a best practice pipeline that contains the estimation of contamination level, mouse-read filtration and variant filtration. The authors finally claimed that the inclusion of mouse cells in patient-derived models hinders genomic analysis and should be addressed carefully and the suggested guidelines improve the robustness and maximize the utility of genomic analysis of these models.

This work is an important patch in cancer research and cancer therapy, especially in the scenario where the downstream application is for conducting personalized treatment to patients. Here are a few issues that the authors need to address and/or discuss before being considered for publication.

Major issues:

1.    In the Background section, the statements that discuss the motivation of this study need more references to support. For example, in Page 2 line 40-42, the authors stated “Indeed, research has shown that resected PDX samples can harbor up to 80% murine DNA without a mistake in the separation process”, more references are needed here and what situation could 80% of murine DNA be mixed into the sequencing library need to be discussed? Same problem applies to the following in vitro statements; and in the last sentence, “Accordingly, researchers have lobbied continuing demands for efficient tools with which to deconvolute or remove murine effects in genomic analyses of PDM models”, again reference.

*Answer to Q1 - We appreciate the reviewer’s advice, and we’ve added more references that support the context [**1-4**], which can be found in the revised manuscript. In particular, Lin et al. mentioned that ‘During expansion of human cancer xenografts in mice, human stromal cells are largely replaced by mouse stromal cells, resulting in mixed species samples.’, and they have reported maximum 73% mouse DNA contaminated xenograft sample out of 93 xenografts [**4**]. About the sentence “Accordingly, researchers have lobbied~”, we thought that the existence of >5 computational tools naturally support it. However, we could also add two more references that directly mentioned it. Finally, we added another reference from NIH’s NCI Patient-Derive Models Repository (PDMR) that mentions the model limitation by the dominant amount of murine stroma (70-80%) in the several cancer type PDM models [**5**].*

2.    When conducting the analyses and statistics, read length is set to 100-bp, but according to the current NGS technology, usually 1-10 bp and bp after 60 or 100 contain low quality readout, therefore adding another level of noise to the human-mouse mixture model. The authors need to experiment different read length to justify their findings. This applies to both DNA-seq and RNA-seq, as the former holds the false SNV/SNP calling and the latter trades off the reads mappability with accuracy.

*Answer to Q2 - First of all, we do agree with the fact that if the base quality at the start and end of the read drops, and it can act as another noise alongside to the HAMA. To rule out this, we performed quality control on all raw-read files at the beginning of the analysis, and used the only samples that showed a reliable base quality. That is the reason we discarded the BALB/c no.2 mouse sample.*

*Second, we accepted the reviewer’s suggestion and performed read trimming on all QC passed raw files using ‘Trimmomatic’ tool. This tool is known to be one of the best trimming performance in terms of mappability [**6**]. We set the ‘max info target length’ parameter to 40, and ‘max info strictness’ parameter to 0.4, suggested by the developer [**7**].*

Figure R1 FASTQC results after applying read trimming with trimmomatic.

*Fig. R1 shows the result of FASTQC before and after applying read trimming. After trimming with Trimmomatic, it was confirmed that the quality score at the end of the reads slightly increased. However, even before performing the read trimming, the middle quartile at the end of the reads exceeds the Quality Score Q30 (this corresponds to 0.1% of base-call error probability). This quality score is considered sufficient to make no false call noise [**8**].*

*Third, and also importantly, the most of widely used aligners are already designed to calculate the alignment score considering the base quality drop at both ends. For the end of the reads that do not pass the quality threshold, soft-clipping is applied (by default) to remove those sequences from the alignment. This is mentioned by Heng Li, the developer of BWA in the mailing list, “You don’t need to do quality trimming with bwa-mem. BWA-backtrack requires reads to be mapped in full length. Low-quality tail may greatly affect its sensitivity. Bwa-mem largely does local alignment. If a tail cannot be mapped well, it will be soft clipped.” (*[*https://sourceforge.net/p/bio-bwa/mailman/message/32030167/*](https://sourceforge.net/p/bio-bwa/mailman/message/32030167/)*). This also applies to other aligners such as Bowtie2. Liu et al even claimed that trimming process is unnecessary because they cause false variant calls [**9**]. We designed the experiments assuming the contamination of mouse reads that can occur in the conventional NGS analysis process (as many researchers will use it), which does not include the trimming process.*

3.    As the author stated that up to 80% of murine DNA could be mixed into the human reads, then why the noise level are only tested on up to 50%? 70% 80%, and even 90% noise level need to be tested as well to include extreme cases.

*Answer to Q3 - We agreed with the reviewer’s suggestion and added 80% contaminated samples to the dataset, and added the analysis results to Fig. 2F, Fig. 3B, Fig. 4, and Supplementary Fig. S3. The overall trend that occurred with increasing contamination was still valid at 80%, but the results of somatic mutation call shown in Fig. 4D are out of trend. This may be due to the decrease in the total number of calls as more than half of the reads are filtered out.*

4.    Specifically, how could an end user estimate the contamination level need to be discussed in detail?

*Answer to Q4 - Users can collect the VAFs of predefined HAMA positions in their samples. The HAMA positions are included in the Additional Files of the paper. The calculation of VAFs can be done with mpileup (from Picard toolkit), or CollectAlleicCounts (from GATK toolkit). The median of the HAMA VAFs is then calculated to finally estimate the contamination rate. We added this to the revised manuscript.*

*In addition, to support this process to general users who are not familiar to Bioinformatics toolkits, we created a GitHub page that contains all the required resources and python scripts for this. (*[*https://github.com/ShockYoung/BestPractice_for_PDMseq*](https://github.com/ShockYoung/BestPractice_for_PDMseq)*).*

5.    In cancer genome, the occurrence of somatic SNV is not totally random but somehow shaped by its 3D genome environment (refer to the Somatic Co-mutation Hotspot (SCH) theory in [https://www.nature.com/arThticles/srep35270](https://www.nature.com/articles/srep35270)). How could this kind of prior knowledge affect or help the filtration step need to be discussed.
*Answer to Q5 - We appreciate the reviewer’s suggestion. After reviewing the Somatic Co-mutation Hotspot (SCH) theory from the provided paper, we concluded that this can be useful in the variant filtration step if there is a sufficient number of SCHs in the database. It should be also noted that the SCHs are observed at the inter-gene and inter-chromatin levels, short reads (~100bp) sequencing might not be very useful at the current stage. However, this can be particularly useful with chromosome conformation capture such as Hi-C. We discussed it in Discussion with referring to the specific paper.*

6.    Essentially, the wet lab tracer technology that might be able to provide single-cell level cell origin information (either murine or human cancer) is the game changer for this sub-area. The current advances for this part need to be included in the Discussion section of this work.

*Answer to Q6 - We again appreciate the suggestion. The wet-lab tracer technologies such as FACS or IMS at the pre-sequencing step would be the most reliable murine genome filtering method. While current barriers to the application of those technologies lie in the time, cost and labors, the “game changing” technologies will be within a good cost-efficient method for the PDM analysis. We also discussed it with the SCH-based filtration in the newly added paragraph in Discussion.*

Minor issues:
1.    The BWA parameters are set default? What is the default? And how allowing different levels of gap and mismatch could affect the analyses need to be discussed.

*Answer to q1 - We are sorry for the lack of detailed description about the BWA-MEM settings. More detailed information has been added to Methods section of the manuscript. The default parameters of the BWA-MEM are as follows.*

*Match reward = 1*

*Mismatch penalty = 4*

*Gap open penalty = 6*

*Gap extend penalty = 1*

*If BWA-MEM is executed without the optional argument, the alignment process is performed without changing these parameters. The use of the default parameter setting is recommended the GATK Best practices (*[*https://software.broadinstitute.org/gatk/best-practices/workflow?id=11165*](https://software.broadinstitute.org/gatk/best-practices/workflow?id=11165)*) and NIH’s GDC Documentation (*[*https://docs.gdc.cancer.gov/Data/Bioinformatics_Pipelines/DNA_Seq_Variant_Calling_Pipeline/*](https://docs.gdc.cancer.gov/Data/Bioinformatics_Pipelines/DNA_Seq_Variant_Calling_Pipeline/)*).*

*A typical mouse originated read (including unmapped reads) contains an average of 4 bp mismatches to the Human Reference. Regarding this increased discrepancy, an increase of mismatch penalty may increase the filtration of mouse read ab initio before the analysis pipeline. This also applies to the indels by increasing the gap extend penalty.*

*Nevertheless, the adjustment of mismatch and gap penalty can also affect the alignment of human originated read, and the overall alignment scores and distributions. As most of the post-alignment pipelines are optimized with the default alignment parameters, this deviation would lead to false positive variants [**10**]. We also assume that most of the NGS data for PDM analysis pipeline would be aligned with default parameters, so our assessment will give the best guideline based on the alignment files. Therefore, we performed alignment process with default parameters and would like to keep it at the final practice.*

2.    The color scheme in Figure 5D is not very distinguishing, richer contract color scheme is advised.

*Answer to q2 - We appreciate the reviewer’s advice. We changed the color scheme for better visibility.*

3.    The formulas in this manuscript need to be presented in a more mathematical way rather than descriptive.

*Answers to q3 – Following the reviewer’s advice, the formulas in the Methods part have been revised (25th line on page 15, 3rd line on page 16), and the revised formulas have been added to the Results part as well (22nd and 27th line on page 6, 4th line on page 7).*

Reviewer #2: The authors of the study attempt to address the important problem of removing mouse reads that often contaminate human reads in studies involving patient-derived xenografts. Since mouse DNA fragments could map quite comfortably to highly conserved regions in the human genome, failing to remove mouse DNA could result in false calls and false interpretations by mutations detection methods. This makes genetic analysis of patient derived xenografts more tedious than original tumor samples. Here, the authors benchmark eight tools that filter mouse reads using simulated datasets with specific mouse contamination levels and subsequently provide best practices for future studies.

Here are some of the comments about the paper,
The authors describe the results using human lung cancer cells from TCGA consortium. They use reads from mouse liver to simulate the contamination. Q1) However, they do not discuss their reasoning behind the choices for these specific cell types. Given the tremendous heterogeneity associated with cancer cells, the authors should discuss if their results would generalize to all types of cancer cells, regardless of the tissue. Q2) Since the authors ran multiple tools to benchmark them, they should comment on the computational speed and requirements for the tools they compared. Their experimental design to simulate data by mixing mouse reads with human reads with mouse cells amounting to 5, 10, 20 or 50% of the total reads with three replicates is robust. Overall a good paper.

**We divided this reviewers question into two parts as noted as Q1 and Q2.*

**Answer to Q1** - We appreciate the valuable comments and the good overall evaluation of our manuscript. Firstly, we do agree with the reviewer that we need to clarify the reason of the choices of cancer type. When designing *in-silico* contaminated samples, we used human tumor data rather than normal to reflect the actual PDX analysis as much as possible. According to the Mouse Tumor Biology (MTB) Database (<http://tumor.informatics.jax.org/mtbwi/>), lung cancer is one of the most frequently registered cancer types in PDX-based studies along with ‘colon and other intestine cancer’. In addition, we assumed that genomic DNA sequences would not be tissue- or cancer-specific except a few cancer-type specific driver mutations. So, we expect that the rendered best-practice pipeline can be generally useful for any cancer types. As suggested by the reviewer, the discussions about cancer type and generality have been added in the revised manuscript, 12^th^ line on page 12.

**Answer to Q2** - As suggested by the reviewer, a table of system requirements and computation speeds of all filtering methods has been added to supplementary information (shown here as Table R1).

| Tools | Input file type | Operating System | Memory Requirement | Programming Language | Library Dependencies | Running Time^*^ |
| --- | --- | --- | --- | --- | --- | --- |
| BBsplit | FASTQ | Linux, MAC, Windows | 32G | JAVA | - | ~ 6 hours |
| Xenome | FASTQ | Linux | 32G | C++ | g++, cmake, libboost-all-dev, pandoc, zlib1g-dev, libbz2-dev, libsqlite3-dev | ~ 72 hours |
| Bamcmp | SAM/BAM | Linux | 4G | C++ | htslib | ~ 2 hours |
| Disambiguate | SAM/BAM | Linux | 4G | C++, Python | zlib, Bamtools C++ API / Pysam | ~ 5 hours |
| XenofilteR | SAM/BAM | Linux, Mac, Windows | 32G | R | Rsamtools, GenomicAlignments, BiocParallel, futile.logger | ~ 1 hours |
| ConcatRef | SAM/BAM | Linux, Mac | 4G | Bash | - | ~ 1 hour |
| DualRef | SAM/BAM | Linux, Mac | 4G | Bash | - | ~ 1 hour |

Table R1 A Summary of the system requirements of filtering methods.

*Please note that the running time is measured only the actual filtering step of each method. The time required to prepare the input for each method can vary.

Reviewer #3: Patient-derived models (PDM) are popular in clinical cancer research. The inevitable inclusion of mouse genome in patient-derived models is a remaining concern in genomic analysis due to the genomic similarity in coding regions between humans and mice. Multiple tools and strategies have been developed for mouse-read filtration. In this manuscript, the authors constructed a benchmark to demonstrate the impact of mouse reads in genomic analysis and to assess the performance of eight filtering methods in terms of mouse-read filtration and reduction of mouse-specific alleles. Furthermore, the authors gave a practice pipeline for variant analysis when analyzing human genome data contaminated by mouse genome. Overall, this is some useful benchmark study. I have several comments as described below.

1.    Although the results in Fig. S6 showed that the average of false variants by “filtering method only” was higher than the average of false variants by “filtering method plus Lenient Blacklist” or by “filtering method plus Strict Blacklist” (2.9 VS 0.8 or 2.9 VS 0.7), the average of loss of true variants by “filtering method only” was lower than the average of loss of true variants by “filtering method plus Lenient Blacklist” or by “filtering method plus Strict Blacklist” (0 VS 4.8 or 0 VS 11.5). Therefore, it is hard to claim “filtering method plus Lenient Blacklist” or “filtering method plus Strict Blacklist” performs better than “filtering method only in calling variants”. 
Furthermore, given the small numbers (false variants and true variants), the existing methods (filtering method only) seem working very well. Do we really need the two suggested pipelines?

**Answer to Q1 -** We appreciate the important issue raised by the reviewer. This is a delicate problem and we had a lot of discussions among the authors. The basis of our decision to include the blacklists (lenient and strict) was based on the ratios (not the absolute numbers) of further filtered false variants (to the total number of false variants) and the lost true variants (to the total number of true variants). As shown in the Table R2, the number of lost true variants (4.79 and 12.9 in lenient and strict blacklisting, respectively) corresponds to 0.4% and 1.1% of the total, while the number of the further filtered variants (2.06 and 2.14 in lenient and strict) corresponds to 72.0% and 74.8% of the total.

Nevertheless, as the reviewer implied, the preference between higher sensitivity and specificity is always study dependent. Sometimes, the loss of 1% true variant cannot be tolerated in some studies to find functional/pathogenic variants. In this case, the blacklisting is not efficient. We concluded that the only way to resolve this problem is to provide sufficient information to users in deciding to or not to include the blacklisting step. Therefore, we newly added discussion with the table in the Supplementary Table S3 and modified the best practice pipeline by marking the blacklisting step as optional.

| **Tools** | **Human-derived variants** | **lost human variants with** | | | | **Mouse-derived variants** | **filtered mouse variants with** | | | |
| --- | --- | --- | --- | --- | --- | --- | --- | --- | --- | --- |
|  |  | **Lenient**  **Blacklisting (%)** | | **Strict**  **Blacklisting (%)** | |  | **Lenient**  **Blacklisting (%)** | | **Strict**  **Blacklisting (%)** | |
| **BBsplit** | 1274.0 | 5.9 | (0.5%) | 15.2 | (1.2%) | 3.2 | 2.4 | (75.0%) | 2.7 | (84.4%) |
| **Bamcmp** | 1273.6 | 6.3 | (0.5%) | 15.7 | (1.2%) | 4.6 | 2.8 | (61.5%) | 2.9 | (63.7%) |
| **ConcatRef** | 1272.7 | 5.1 | (0.4%) | 14.5 | (1.1%) | 2.7 | 1.7 | (64.2%) | 1.8 | (66.0%) |
| **Disambiguate** | 1274.5 | 6.4 | (0.5%) | 15.7 | (1.2%) | 4.1 | 2.7 | (65.4%) | 2.7 | (66.7%) |
| **DualRef-S** | 864.2 | 1.1 | (0.1%) | 4.8 | (0.6%) | 1.6 | 1.6 | (100.0%) | 1.6 | (100.0%) |
| **XenofilteR** | 1262.7 | 5.7 | (0.4%) | 14.2 | (1.1%) | 2.0 | 1.6 | (80.0%) | 1.6 | (80.0%) |
| **Xenome** | 1263.2 | 3.2 | (0.3%) | 10.3 | (0.8%) | 2.0 | 1.7 | (84.6%) | 1.7 | (87.2%) |
| **Average** | 1212.11 | 4.79 | (0.4%) | 12.9 | (1.1%) | 2.86 | 2.06 | (72.0%) | 2.14 | (74.8%) |

Table R2 The efficiency of strict and lenient blacklisting

2.    Is the estimate of global contamination level reliable?  1) the four Hf data points in Fig 2F are means of repeated samples. How fluctuating are those Hf from a single sample?  In realistic experiments, you have only one sample.

**Answer to Q2** - Again, we appreciate the reviewer’s comment. After discussing with authors, we found that the current figure is not efficient to show the reliability of the estimation and finally changed it to a different plot (revised Figure 3F, figure also shown below). The current plot showed that the relationship between the median HAMA VAFs (*H_f_*) and the global contamination level. As the reviewer pointed out, this explained how we found the coefficient between the two variables (HAMA VAF and global contamination level) and not how much the estimation is reliable. Therefore, we added a new plot that shows the relationship between the true contamination levels and the estimated contamination levels (based on the inferred coefficient) in 150 benchmark samples (5 strains, 3 downsampling replicates to the 2 human samples, 5 different contamination levels from 5% to 80%; assessment of 80% contamination is newly added based on other reviewer’s suggestion). Please note that this is exactly how the contamination level estimation is applied to any further PDM samples from users. The new results show that our simple estimation can predict the global contamination level within an acceptable error range (0.4% to 2%, 95% CI); a slight under estimation in <50% contamination and overestimation in 80% is observed, nevertheless. Although we have some ideas for better and more sophisticated methods including separation of hetero and homo HAMAs and use of probabilistic fitting models, we think that development of an independent method is beyond the scope of this paper. Therefore, we would like to leave them as a further study if the reviewers agree.

3.    In page 8 line 57, “(Table 1, see Methods for detailed benchmark procedures).” Table 1 is missing.

**Answer to Q3** - We corrected the manuscript as the reviewer pointed out. We appreciate the reviewer’s careful assessment.

4.    In Fig. S6, the x-labels, “Filtering method + Lenient Blacklisting” and “Filtering method + Strict Blacklisting” are misplaced.
**Answer to Q4** - We corrected the label. Again, we appreciate the careful note.

We thank all the reviewers for their careful reading of the manuscript and valuable comments.

**References**

1.	Chivukula IV, Ramskold D, Storvall H, Anderberg C, Jin S, Mamaeva V, Sahlgren C, Pietras K, Sandberg R, Lendahl U: **Decoding breast cancer tissue-stroma interactions using species-specific sequencing.** *Breast Cancer Res* 2015, **17:**109.

2.	Ben-David U, Ha G, Tseng YY, Greenwald NF, Oh C, Shih J, McFarland JM, Wong B, Boehm JS, Beroukhim R, Golub TR: **Patient-derived xenografts undergo mouse-specific tumor evolution.** *Nat Genet* 2017, **49:**1567-1575.

3.	Farren M, Weston S, Brown H, Broadbent N, Powell S, Shaw R, Smith NR, Inglis R, Graham A, Ashton S, et al: **Expression of stromal genes associated with the angiogenic response are not differentiated between human tumour xenografts with divergent vascular morphologies.** *Angiogenesis* 2012, **15:**555-568.

4.	Lin MT, Tseng LH, Kamiyama H, Kamiyama M, Lim P, Hidalgo M, Wheelan S, Eshleman J: **Quantifying the relative amount of mouse and human DNA in cancer xenografts using species-specific variation in gene length.** *Biotechniques* 2010, **48:**211-218.

5.	**NCI Patient-Derived Models Repository** [<https://pdmr.cancer.gov/models/limitations_caveats.htm>]

6.	Del Fabbro C, Scalabrin S, Morgante M, Giorgi FM: **An extensive evaluation of read trimming effects on Illumina NGS data analysis.** *PLoS One* 2013, **8:**e85024.

7.	Bolger AM, Lohse M, Usadel B: **Trimmomatic: a flexible trimmer for Illumina sequence data.** *Bioinformatics* 2014, **30:**2114-2120.

8.	**Quality Scores for Next-Generation Sequencing** [<https://www.illumina.com/documents/products/technotes/technote_Q-Scores.pdf>]

9.	Liu Q, Guo Y, Li J, Long J, Zhang B, Shyr Y: **Steps to ensure accuracy in genotype and SNP calling from Illumina sequencing data.** *BMC Genomics* 2012, **13 Suppl 8:**S8.

10.	Li H, Homer N: **A survey of sequence alignment algorithms for next-generation sequencing.** *Brief Bioinform* 2010, **11:**473-483.
